# Beneficial effects of dietary supplementation with green tea catechins and cocoa flavanols on aging-related regressive changes in the mouse neuromuscular system

**DOI:** 10.18632/aging.203336

**Published:** 2021-07-28

**Authors:** Sílvia Gras, Alba Blasco, Guillem Mòdol-Caballero, Olga Tarabal, Anna Casanovas, Lídia Piedrafita, Alejandro Barranco, Tapas Das, Ricardo Rueda, Suzette L. Pereira, Xavier Navarro, Josep E. Esquerda, Jordi Calderó

**Affiliations:** 1 Unitat de Neurobiologia Cel·lular, Departament de Medicina Experimental, Facultat de Medicina, Universitat de Lleida and Institut de Recerca Biomèdica de Lleida (IRBLleida), Lleida, Spain; 2 Grup de Neuroplasticitat i Regeneració, Institut de Neurociències, Departament de Biologia Cellular, Fisiologia i Immunologia, Universitat Autònoma de Barcelona and CIBERNED, Bellaterra, Spain; 3 Department of Biochemistry and Molecular Biology II, School of Pharmacy, University of Granada, Granada, Spain; 4 Abbott Nutrition, Research and Development, Columbus, OH 43215, USA; 5 Abbott Nutrition, Research and Development, Granada, Spain

**Keywords:** sarcopenia, green tea, neuromuscular system, aging, cocoa

## Abstract

Besides skeletal muscle wasting, sarcopenia entails morphological and molecular changes in distinct components of the neuromuscular system, including spinal cord motoneurons (MNs) and neuromuscular junctions (NMJs); moreover, noticeable microgliosis has also been observed around aged MNs. Here we examined the impact of two flavonoid-enriched diets containing either green tea extract (GTE) catechins or cocoa flavanols on age-associated regressive changes in the neuromuscular system of C57BL/6J mice. Compared to control mice, GTE- and cocoa-supplementation significantly improved the survival rate of mice, reduced the proportion of fibers with lipofuscin aggregates and central nuclei, and increased the density of satellite cells in skeletal muscles. Additionally, both supplements significantly augmented the number of innervated NMJs and their degree of maturity compared to controls. GTE, but not cocoa, prominently increased the density of VAChT and VGluT2 afferent synapses on MNs, which were lost in control aged spinal cords; conversely, cocoa, but not GTE, significantly augmented the proportion of VGluT1 afferent synapses on aged MNs. Moreover, GTE, but not cocoa, reduced aging-associated microgliosis and increased the proportion of neuroprotective microglial phenotypes. Our data indicate that certain plant flavonoids may be beneficial in the nutritional management of age-related deterioration of the neuromuscular system.

## INTRODUCTION

Sarcopenia, the progressive loss of skeletal muscle mass and function with age, is considered the main causative factor of the physical performance decline in the elderly. The compromised muscular function associated to sarcopenia has a negative impact on the life quality of older adults and increases the risk for adverse health outcomes including disability, fall-associated injuries, morbidity, and mortality [1]. All these aspects stress healthcare systems, increasing the care needs of older people and their costs. With a prevalence of 8-40% in people over 60 years, depending on studies [2, 3], sarcopenia constitutes a critical challenge in our aging society. Starting in the third decade of life, humans experience a gradual deterioration of muscle power, and over the age of 60 a decline of about 3% of muscle mass per year has been reported to occur in most of individuals [3, 4]. Different mechanisms have been identified to contribute to the loss of muscle mass with age. In fact, sarcopenia in the elderly is presently considered a complex multifactorial condition which involves intrinsic and extrinsic causative factors (reviewed by [5]); among of them, chronic inflammation, metabolic and endocrine alterations, poor nutrition, mitochondrial dysfunction, oxidative damage, and neurogenic factors, appear to play an important role in sarcopenia.

Accompanying the skeletal muscle wasting, a plethora of structural and functional changes has been found at advanced ages in distinct components of the neuromuscular system, including spinal cord motoneurons (MNs), dorsal root ganglion (DRG) sensory neurons, and neuromuscular junctions (NMJs) ([6], see also [5, 7–9] as reviews). Thus, studies performed in both animals and humans have reported that, with aging, MNs become dysfunctional, show a marked depletion of afferent nerve terminals and, some of them, exhibit signs of degeneration. Prominent gliosis, involving both microglia and astroglia, have also been found in the spinal cord around aged MNs. In this vein, we have recently reported a marked increase in the microglial and astroglial pro-inflammatory phenotypes (M1 and A1, respectively) in the spinal cord of aged mice to the detriment of anti-inflammatory and neuroprotective (M2 and A2) glial subpopulations [6]. Additionally, with age, NMJs appear to be remodeled and exhibit marked changes in their pre- and postsynaptic compartments in association with the loss of motor units, reactive nerve sprouting and cycles of muscle denervation/reinnervation. Aging is also accompanied by alterations in DRG sensory neurons in form of changes in the expression of different neuropeptides and atrophy of both proprioceptive and nociceptive neuronal populations [6]. However, on the whole, the primary contribution of such defects in the skeletal muscle deterioration with aging is not yet well stablished. In this regard, the fact that the age-associated NMJ disruption is a secondary process resulting from intrinsic defects in the skeletal muscle cannot be excluded.

Age-related decline in muscle function and motor activity is susceptible to be ameliorated by lifestyle interventions such as exercise and/or nutritional strategies [10–15]. Caloric restriction, based on a diet low in calories, has been shown to attenuate aging sarcopenia in various species by acting at different levels of the skeletal muscle. Thus, a line of evidence indicates that a moderate reduction in caloric intake, alone or combined with exercise training, is able to mitigate mitochondrial alterations and production of reactive oxygen species, modulate autophagy, enhance the endogenous myofiber repair and counteract the increase in proapoptotic signals observed in aged skeletal muscles [16–20]. Caloric restriction has also been reported to ameliorate age-related changes in rodent NMJs and to prevent MN and motor axon degeneration found to occur with aging [11, 21]. In a similar way, some dietary supplements have been shown to counteract age-related changes that contribute to neuromuscular dysfunction (reviewed by [12]). Thus, several nutritional interventions, involving supplementations with vitamin D, omega-3 fatty acids, creatine, β-hydroxy-β-methybutyrate, or dietary phospholipids, have been reported to be beneficial in promoting healthy neuromuscular aging throughout different mechanisms; these include: *a)* myotrophic, neurotrophic and anti-inflammatory actions; *b)* regulation of pathways involved in mitochondrial function and reactive oxygen species production; *c)* inhibition of muscle proteolysis; and *d)* improvement of cellular bioenergetics.

Plant flavonoids have gained particular attention as dietary compounds for keeping good health and preventing a number of diseases, particularly cardiac disorders and cancer [22]. Several studies have reported the effects of polyphenolic-flavonoids-containing supplements in the attenuation of oxidative stress and muscle damage, particularly after intensive strength and resistance exercise [23–28]. In this regard, previous research has shown the benefits of polyphenols contained in green tea and its extracts on the endurance capacity by preventing muscle impairment in patients with Duchenne muscular dystrophy [29] and mouse models of this disease [30–32], and also in aging [33, 34] and immobilization-induced muscle loss [35, 36]. Green tea extract (GTE) is enriched in flavonoids known as catechins (including catechin, epicatechin, gallocatechin, epigallocatechin, epicatechin gallate and, predominantly, epigallocatechin gallate [EGCg]), which have strong antioxidant and anti-inflammatory properties [37, 38]. A class of flavonoids known as flavanols are also the main constituents of cocoa beans and their derivatives. Thus, cocoa products are high in non-esterified monomers of the flavanols catechin and epicatechin, and also proanthocyanidins [39]. Among of these compounds, epicatechin represents about 35% of the total content of polyphenols in cocoa beans, and appears to be the main responsible for the beneficial effects of dietary cocoa. In fact, cocoa polyphenols have been shown to exert diverse physiological activities as antioxidants, cardiovascular protectors, immunomodulators, anti-inflammatory agents and potential tumor protectors [40–44]. Epicatechin has been reported to promote neuroprotection *in vitro* against oxidative stress [45]. Moreover, *in vivo* studies in mice have found that an epicatechin dietary supplementation enhances the oxidative capacity of the skeletal muscle and improves the survival rate of animals and the physical performance impairment occurring with aging [46, 47]. Despite the body of reported data on the beneficial actions of dietary polyphenols on physical activity and exercise performance, there is little work examining the effects of these compounds on the structural and molecular organization of the neuromuscular system. In fact, most of the previous analyses have been focused on flavonoid actions in diverse skeletal muscle parameters with very little information on their effects on the neural elements of the neuromuscular system.

In the present study, we tested the impact of flavonoid-enriched diets containing either GTE catechins or cocoa flavanols, on the age-related deterioration of the neuromuscular system. Our main goal was to explore the potential benefits of these flavonoids on age-associated alterations of the spinal cord MNs and glia, DRG sensory neurons, motor nerves, NMJs, and skeletal muscles, and whether these interventions can mitigate the age-related motor decline in a relevant mouse model [6].

## RESULTS

### GTE and cocoa flavanols improve the survival rate of mice

**Figure 1:**
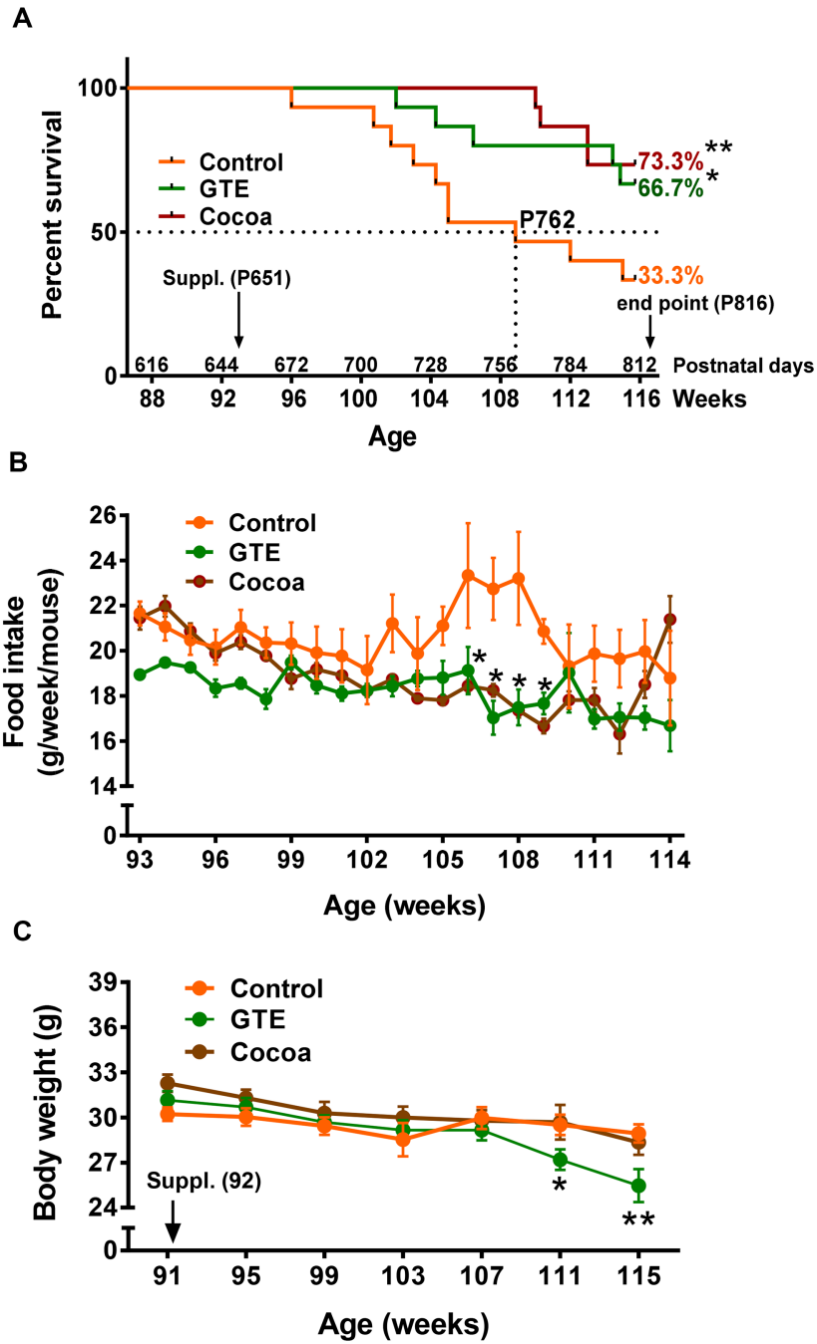
**GTE and cocoa supplementations improve mouse survival rate.** (**A**) Survival rate of mice from control, GTE and cocoa groups; the standard (AIN-93M) diet supplemented with either cocoa or GTE significantly increased the percentage of mice that were alive at the endpoint of experiment compared with control animals. (**B**) Food intake (expressed as g of food per week and mouse) was reduced in animals fed with the GTE- or cocoa-supplemented diets, this reduction being statistically significant in a period ranging from 105-109 weeks of age. (**C**) Body weight (g) of animals from the three experimental groups; compared to control mice, no significant changes in body weight were found in animals from cocoa group; a significant reduction in weight was, however, observed in GTE-supplemented mice. Data are shown as the mean ± SEM (number of animals per group: 91 weeks of age, n = 15 in all groups; 107 weeks, control n = 8, GTE n = 13; cocoa n = 15; 114 weeks, control n = 6, GTE n = 12, cocoa n = 11); *p < 0.05 and **p < 0.01 vs. control (Gehan-Breslow-Wilcoxon test, in (**A**) and multiple *t*-test (Bonferroni correction), in (**B**, **C**).

Starting at age of 92 weeks (21 months), mice were fed with a standard (AIN-93M) diet (control group) or AIN-93M diet supplemented with either GTE (GTE group) or cocoa flavanols (cocoa group). All diets were maintained until week 116 (27 months), the selected endpoint in which animals were euthanized for tissue sampling. Daily food intake was 3 g/d and average body weight was ~30 g. At endpoint, animals from GTE group and cocoa group exhibited a significant higher survival rate (~66%, GTE group; ~73%, cocoa group, *p* < 0.05 and *p* < 0.01 respectively) compared to control mice (~33%; Gehan-Breslow-Wilcoxon test) (Figure 1A). In comparison with control group mice, those from GTE and cocoa groups had a reduction in food intake between 105 and 109 weeks of age (Figure 1B). No significant differences in body weight were observed between animals of control and cocoa groups; however, mice fed with GTE-enriched diet exhibited, at 111-115 weeks of age, significantly reduced body weights compared with control animals (Figure 1C).

### GTE or cocoa flavanols ameliorate the aging degree and degeneration rate of skeletal muscle fibers of aged mice

**Figure 2:**
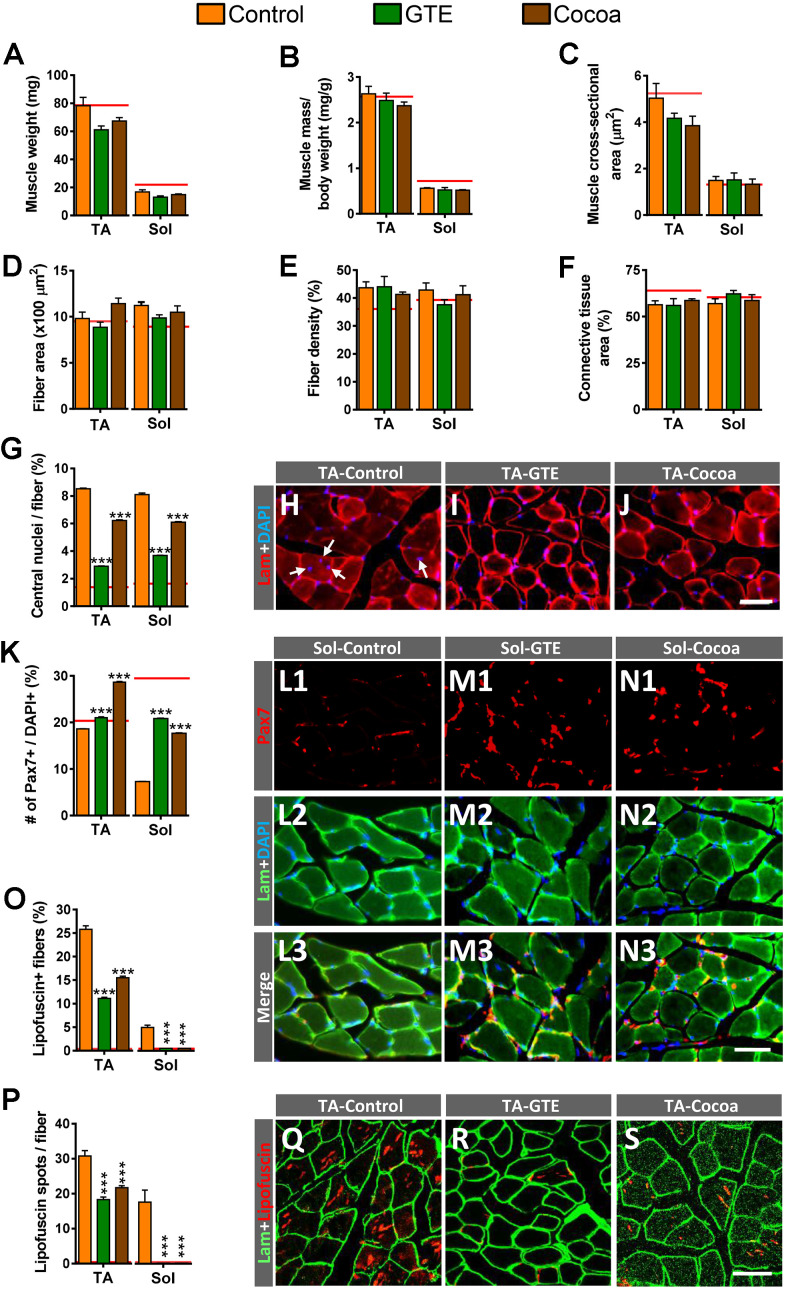
**Impact of GTE- and cocoa-supplemented diets on aging-associated changes in TA and Sol muscles of mice.** (**A**–**C**) Muscle wet weight (in mg, **A**), muscle weight relative to body weight (expressed in mg/g, **B**) and muscle cross-sectional area (in μm^2^, **C**) are shown. (**D**) Average myofiber size (cross-sectional area in μm^2^). (**E**, **F**) Density of myofibers (**E**) and muscular content of connective tissue (**F**), expressed as the percentage of area occupied by either myofibers or connective tissue respect to the total cross-sectional muscle area). (**G**) Proportion of myofibers displaying central nuclei. (**H**–**J**) Representative images of transversal cryosections of TA muscles of control, GTE and cocoa groups (as indicated); sections were double labeled with an antibody against laminin (red) and DAPI (blue) for DNA; arrows in (**H**) indicate central nuclei. (**K**) Percentage of Pax7-immunostained cells (SCs) with respect to DAPI-positive nuclei. (**L1**–**N3**) Representative images of a combined immunolabeling for Pax7 (red) and laminin (green), and DAPI staining (blue) in transversal cryosections of Sol muscles from control, GTE and cocoa groups, as indicated. (**O**, **P**) Percentage of myofibers containing lipofuscin aggregates (**O**), and average number of lipofuscin granules per myofiber (**P**). (**Q**–**S**) Representative images of transversal cryosections of TA muscles from control, GTE and cocoa groups (as indicated) immunolabeled for laminin (green); lipofuscin autofluorescence (red) was excited using 510-560 nm excitation and 590 emission filters. Data in graphs are expressed as the mean ± SEM; the average values of adult muscles found in a previous study [6] were indicated by a red line in each graph for comparative purposes; ***p < 0.001 vs. control (Ctrl), two-way ANOVA, Bonferroni’s *post hoc* test; sample size per condition: (**A**–**C**) = 6-12 muscles and (**D**–**F**) = 3-5 muscles from different mice; (**G**, **K**) = 2500-4000 fibers and (**O**, **P**) = 1200-2200 fibers per muscle from 3-5 animals. Scale bars: 40 μm in (**J**) (valid for **H**, **I**), in N3 (valid for **L1**–**N2**) and **S** (valid for **Q**, **R**).

We next examined the potential effects of the GTE- or cocoa-supplemented diets on the aging-associated histological changes found in skeletal muscles in our previous study [6]. Compared to control group, no significant changes in tibialis anterior (TA) and soleus (Sol) muscle-wet weights, either gross or normalized to body weight (muscle mass/body weight) or cross section area, were found in animals fed with any of the two supplemented diets (Figure 2A–2C), indicating that they are not able to prevent the moderate reduction of muscle mass observed in the course of aging. Moreover, supplemented diets did not either provoke any significant change in the size and density of fibers or the content of connective tissue in aged control muscles (Figure 2D–2F).

We and others have reported that skeletal muscles of aged mice frequently display high proportions of fibers exhibiting centrally located nuclei [6, 11]. This change appears to reflect a regenerative process in muscles in an attempt to replace degenerated myofibers that are lost in the course of aging [48, 49]. We found that, compared to control muscles, those from animals of either GTE or cocoa groups showed a significant reduction in the proportion myofibers displaying central nuclei (Figure 2G–2J). On the other hand, our and other labs have described that aging entails a depletion of muscle satellite cells (SCs) [6, 50–53]. The effects of the supplemented diets on SCs were then examined by immunohistochemistry for paired box protein 7 (Pax7), a paired homeobox transcription factor involved in the proliferation of the muscle precursor cells, in combination with DAPI staining. We found that TA and Sol muscles from animals of GTE and cocoa groups had significantly higher densities of SCs than those from control mice (Figure 2K–2N3).

We wanted to analyze next the effects of the flavonoid-enriched diets on the presence of lipofuscin in aged muscles. Lipofuscin is considered a pigment resulting from an oxidative stress process [54], and is widely seen as a hallmark of aging. Lipofuscin has been shown to accumulate in fibers of aged and dystrophic muscles [6, 55, 56]. As expected from our previous results [6], control aged hindlimb muscles exhibited significant differences in lipofuscin content, with the slow-twitch Sol muscle displaying much lower proportions of the pigment than the fast-twitch TA muscle. GTE- and cocoa-supplemented diets significantly reduced the amount of lipofuscin in muscles by decreasing both the number of myofibers containing the pigment and the number of its aggregates (Figure 2O–2S). Overall, these results suggest that flavonoid-enriched diets are able to alleviate the process of skeletal muscle senescence, which is reflected in the reduction of myofiber degeneration. This would result in a decrease in the amount of newly regenerated myofibers, and in the preservation of SC population due to the decline in the number of these stem cells that engage their myogenic program to replace compromised aged myofibers.

### GTE and cocoa flavanols prevent age-associate alterations in skeletal muscle mitochondria

**Figure 3:**
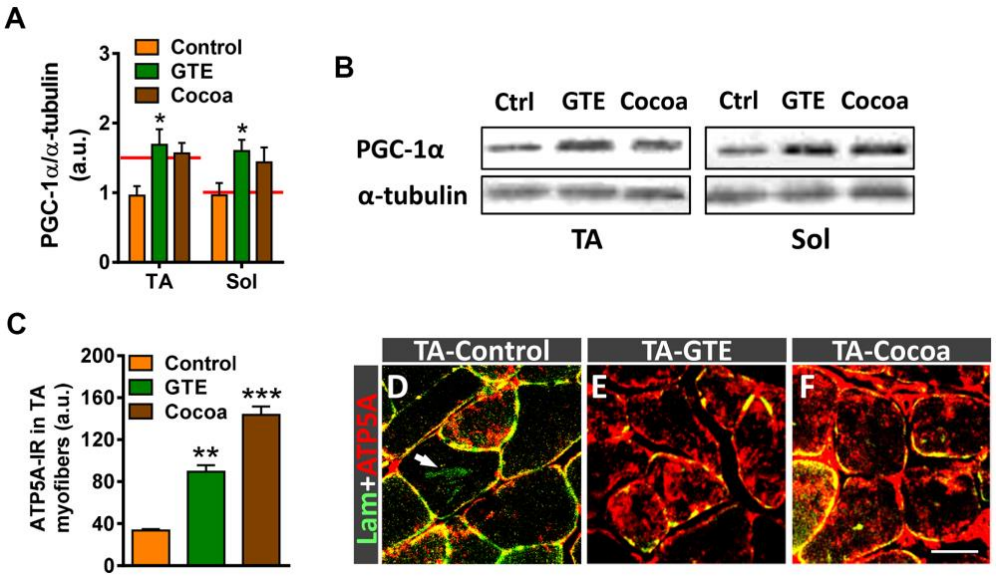
**GTE- and cocoa-supplemented diets partially prevent the decrease in PGC-1α expression and restore mitochondrial depletion occurring muscles in the course of age.** (**A**) Densitometric analysis of changes in PGC-1α levels in muscles from the three experimental conditions; data were normalized to α-tubulin. Bars represent the values (mean ± SEM) of 3 mice per condition from 2 independent western blot analysis; *p < 0.05 *vs.* control, two-way ANOVA (Bonferroni’s post-hoc test); red lines in graph indicate PGC-1α levels found in adult muscles. (**B**) Representative western blots of PGC-1α and α-tubulin (as loading control) proteins in TA and Sol muscles from mice of control (Ctrl), GTE and cocoa groups. (**C**) Quantification of ATP5A-immunoreactivity in TA muscles of control, GTE and cocoa groups; bars represent the values (mean ± SEM) of 3 mice per condition; **p < 0.01 and ***p < 0.001 *vs.* control, one-way ANOVA (Bonferroni’s post-hoc test). (**D**–**F**) Representative images showing a combined immunolabeling for laminin (lam, green) and ATP5A (red) in transversal cryosections of TA muscles from three experimental groups used for quantification, as indicated. Note the overt increase in ATP5A immunostaining after GTE (**E**) and, particularly, cocoa (**F**) supplementation compared to control (**D**). Arrow in (**D**) points out lipofuscin deposition in a myofiber. Scale bar in (**F**): 50 μm (valid for **D**, **E**).

Peroxisome proliferator-activated receptor γ co-coactivator 1α (PGC-1α) is a master regulator of mitochondrial biogenesis and function [57]. PGC-1α appears to play an important role in muscle by regulating myofiber metabolism, autophagy, and the expression of different components of NMJs [58, 59]. Some studies have shown that PGC-1α content declines in muscle with normal aging in parallel with the decrease in mitochondrial function [60–62]. To examine whether GTE and cocoa enhance skeletal muscle metabolism via regulation of PGC-1α, its expression was analyzed by western blotting. Muscle extracts of animals which received either of those supplements resulted in higher levels of PGC-1α protein compared to controls, but only the GTE group achieved statistical significance (Figure 3A, 3B). To examine whether the increased expression of PGC-1α in skeletal muscle induced by GTE and cocoa supplementation was linked to the augmented density of mitochondria in myofibers, we performed immunohistochemistry with an anti-ATP5A antibody, used as a mitochondrial marker. ATP5A immunolabeling was analyzed in the fast-twitch TA muscle, which is predominantly composed of fast myofibers (2A > 2B), which have a glycolytic metabolism and reduced mitochondrial content compared to slow (oxidative) myofibers [6, 63]. Compared to TA muscles of adult mice, those of aged animals showed a significant (~25%) reduction of ATP5A immunostaining in myofibers, indicating that an overt decrease in the number of mitochondria occurs with aging in muscles (ATP5A-immunoreactivity expressed as arbitrary units: adult TA, 94.01 ± 2.10; aged TA, 72.59 ± 1.38; *p* < 0.001, Student’s *t-*test, 100 randomly selected myofibers were analyzed from 4 mice per condition). Muscles of mice fed with either GTE- or cocoa-supplemented diets exhibited significantly higher mitochondrial contents than those of controls (Figure 3C–3F).

### GTE- and cocoa-supplemented diets prevent muscle denervation and improve regressive NMJ alterations occurring with aging

**Figure 4:**
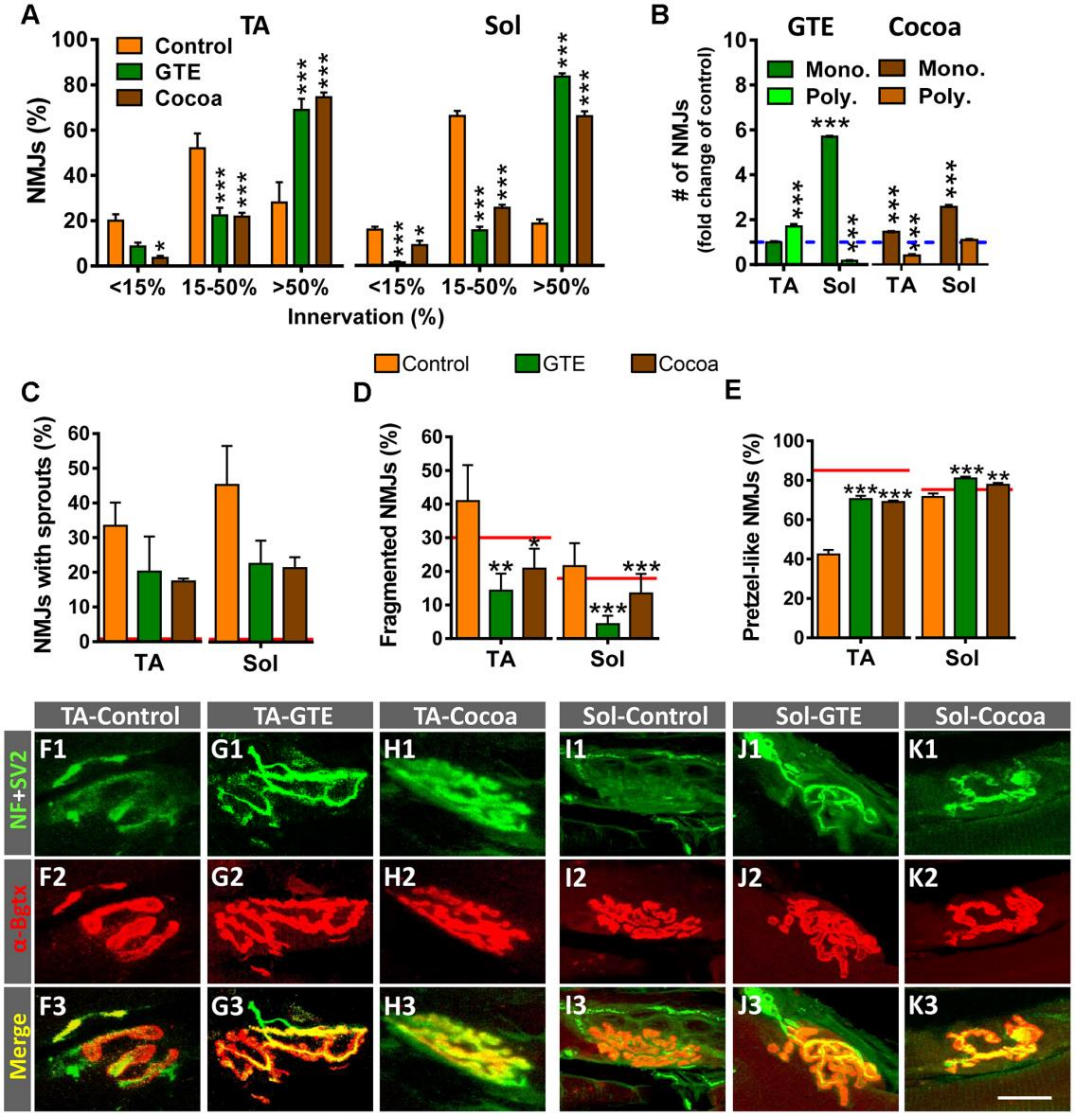
**GTE- and cocoa-supplemented diets prevent aging-associated muscle denervation and regressive morphological alterations in NMJs.** (**A**) Proportion of TA and Sol NMJs displaying different degrees of innervation; quantification was based on the percentage of α-Bgtx-labeled postsynaptic site area covered by SV2-immunostained presynaptic terminals (see Materials and Methods, <15% innervation was considered as denervated). (**B**) Number of NMJs of TA and Sol muscles exhibiting single (mono.) or multiple (poly.) innervation expressed as fold change of control group (blue dashed line). (**C**–**E**) Percentage of NMJs showing terminal axonal sprouts (**C**), fragmented endplates (**D**) and postsynaptic sites exhibiting a pretzel-like appearance (**E**, indicative of high degree of synaptic maturity), in TA and Sol muscles of animals from different experimental groups. Bars in graphs represent the mean ± SEM; sample size: 30-58 (**A**), and 50-85 (**B**–**E**) NMJs per muscle from 3-5 animals per condition; *p < 0.05, **p < 0.01 and ***p < 0.001 vs. control, one or two-way ANOVA, Bonferroni’s *post hoc* test; red lines in (**C**–**E**) indicate values in adult mice previously reported [6]. (**F1**–**K3**) Representative maximal projections of confocal stacks of NMJs of TA (**F1**–**H3**) and Sol (**I1**–**K3**) from mice of control, GTE and cocoa groups (as indicated in panels); muscle sections were stained with antibodies against NF and SV2 (green, for presynaptic nerve terminals), and α-Bgtx (red, for postsynaptic AChR). Scale bar in **K3** = 20 μm (valid for **F1**–**K2**).

Previous studies in rodents have reported that NMJs of skeletal muscles undergo structural and molecular alterations with aging [6, 9, 11, 63–65]. Using the same mouse model, we observed that a significant number of NMJs of aged hindlimb muscles exhibit overt signs of denervation and polyinnervation, increased branching complexity and sprouting of nerve terminals; additionally, postsynaptic sites display a marked fragmentation of endplates, with numerous scattered acetylcholine receptor (AChR) spots which are associated to neurofilament 68 kDa (NF)-positive and vesicular acetylcholine transporter (VAChT)-positive immunostaining of presynaptic structures. Overall, this pattern differs from that we observed in NMJs from mice at early postnatal ages, in which postsynaptic sites display a more homogeneous and undivided morphology, and nerve branching is highly pleomorphic (not shown). All these changes are indicative of a reactive plasticity and neoformation of neuromuscular synapses in the course of aging [6]. We wanted to analyze whether these age-associated changes could be prevented with either a GTE- or a cocoa-supplemented diet. The architecture and innervation of NMJs were examined by double immunostaining for NF and synaptic vesicle protein 2 (SV2, for the presynaptic nerve terminal visualization) combined with α-bungarotoxin (α-Bgtx, for the postsynaptic site identification). The two tested diets significantly reduced the proportion of denervated or partially innervated NMJs found in aged muscles, which was accompanied by an increased amount of NMJs exhibiting a higher degree of innervation (Figure 4A); moreover, the proportion of monoinnervated NMJs was significantly increased with polyinnervated NMJs exhibiting either no changes or a significant reduction in number, except for TA muscle of GTE group mice (Figure 4B). In the same line, the amount of motor axons showing terminal sprouting was reduced in muscles from GTE and cocoa groups compared to controls (Figure 4C); this decline, however, did not attain statistical significance possibly due to sample size and variability between animals. Animals fed with either of the two dietary supplements also displayed significantly decreased proportions of fragmented endplates in muscles (Figure 4D). Additionally, muscles from dietary supplemented animals globally exhibited more “healthy” NMJs, as indicated by the increased number of endplates exhibiting a well-defined “pretzel-like” pattern, which contrasted with the less mature and altered appearance of numerous endplates typically found in control muscles (Figure 4E). Some examples of aged TA and Sol NMJs of control, GTE and cocoa groups are shown in Figure 4F1–4K3.

In our previous work [6] we have shown that aging is associated with changes in the expression of different proteins related to development, plasticity and maintenance of NMJs, such as calcitonin gene-related peptide (CGRP) [66–69], agrin [70], and growth associated protein 43 (GAP-43) [71]. Compared to young mice, the levels of these synaptic molecules in NMJs markedly decline in adult muscles, but are upregulated in those of aged animals [6]. The potential impact of GTE and cocoa supplementations in the content of CGRP, agrin and GAP-43 in aged muscles was examined. In comparison with control NMJs, those from GTE and cocoa groups exhibited a modest, but non-significant increase in CGRP- and agrin-immunoreactivity (Supplementary Figure 1A–1F3). On the other hand, no significant changes in GAP-43-immunostaining at NMJs were found between the three groups (Supplementary Figure 1G–1I3), although the GAP-43 content in the entire muscles determined by western blotting showed a tendency to be decreased in animals that received the dietary supplementations (Supplementary Figure 1J, 1K).

### GTE or cocoa supplements do not prevent the degenerative changes occurring in motor nerves with aging

As previously reported, aging is associated with motor axon degeneration. This can be already noticed in middle-aged mice and include Wallerian-like degenerative changes, myelin spheroidal inclusions, axonal swelling, and reduction of myelin thickness [6, 72]. To analyze whether either GTE- or cocoa-supplemented diets were able to prevent motor nerve degeneration, we examined L4 ventral nerve roots (VRs), which are shaped by the most proximal part of motor axons contributing to the sciatic nerve formation. Neither flavonoids promoted a substantial improvement of structural age-associated motor nerve alterations. No significant changes in the cross-sectional area of VRs, number and diameter (including myelin sheath) of axons, and the proportion of degenerating axons per nerve root were found between the three experimental groups (Supplementary Figure 2A–2F, 2I–2K). We even noticed that, compared with the control group, VRs of animals fed with GTE or cocoa supplements had a higher, although not significant, increase in the number of axons displaying minor signs of degeneration and myelin inclusions. However, roots from cocoa group animals, showed a slightly increase in the number of clusters of small myelinated axons (Supplementary Figure 2G–2K); these could represent immature axons formed by sprouting from healthy ones and suggest the generation of new nerve fibers aimed at replacing those previously degenerated. G-ratio value, as an indicator of the relationship between myelin thickness and axon diameter, did not show significant differences between groups (Supplementary Figure 2L, 2M). However, compared to control VRs, those from GTE and cocoa groups exhibited slightly smaller axonal diameters (excluding the myelin sheath), with values closer to those found in adult mice (Supplementary Figure 2N).

### Dietary supplementation with GTE or cocoa flavanols also ameliorates aging-associated alterations in proximal hindlimb muscles

There is evidence that aging affects differentially distinct muscle groups [73–75]. Our previous data [6] suggest that the degree of muscular wasting with age would be associated not only to muscle location and fiber type composition, but also to specific function and activity of muscles. We have also shown that gracilis (Gra) muscle, which is proximally placed in the hindlimb, exhibited more prominent age-related alterations than distal leg muscles. The effects of GTE- and cocoa-enriched diets were then examined in this proximal muscle. We found that compared to control group, Gra muscles from GTE and cocoa groups displayed significant increased density of Pax7-positive SCs, and reduced proportions of myofibers exhibiting lipofuscin contents (GTE) and decreased numbers of lipofuscin aggregates per myofiber (GTE and cocoa) (Supplementary Figure 3A–3G). Furthermore, Gra muscles from GTE and cocoa groups had a significant reduction in the number of denervated and partially innervated NMJs and an increase in the proportion of those showing a single innervation with respect to control counterparts (Supplementary Figure 4A–E3).

### Effects of dietary supplementations with GTE and cocoa flavanols on MN synaptic afferents of old mice

**Figure 5:**
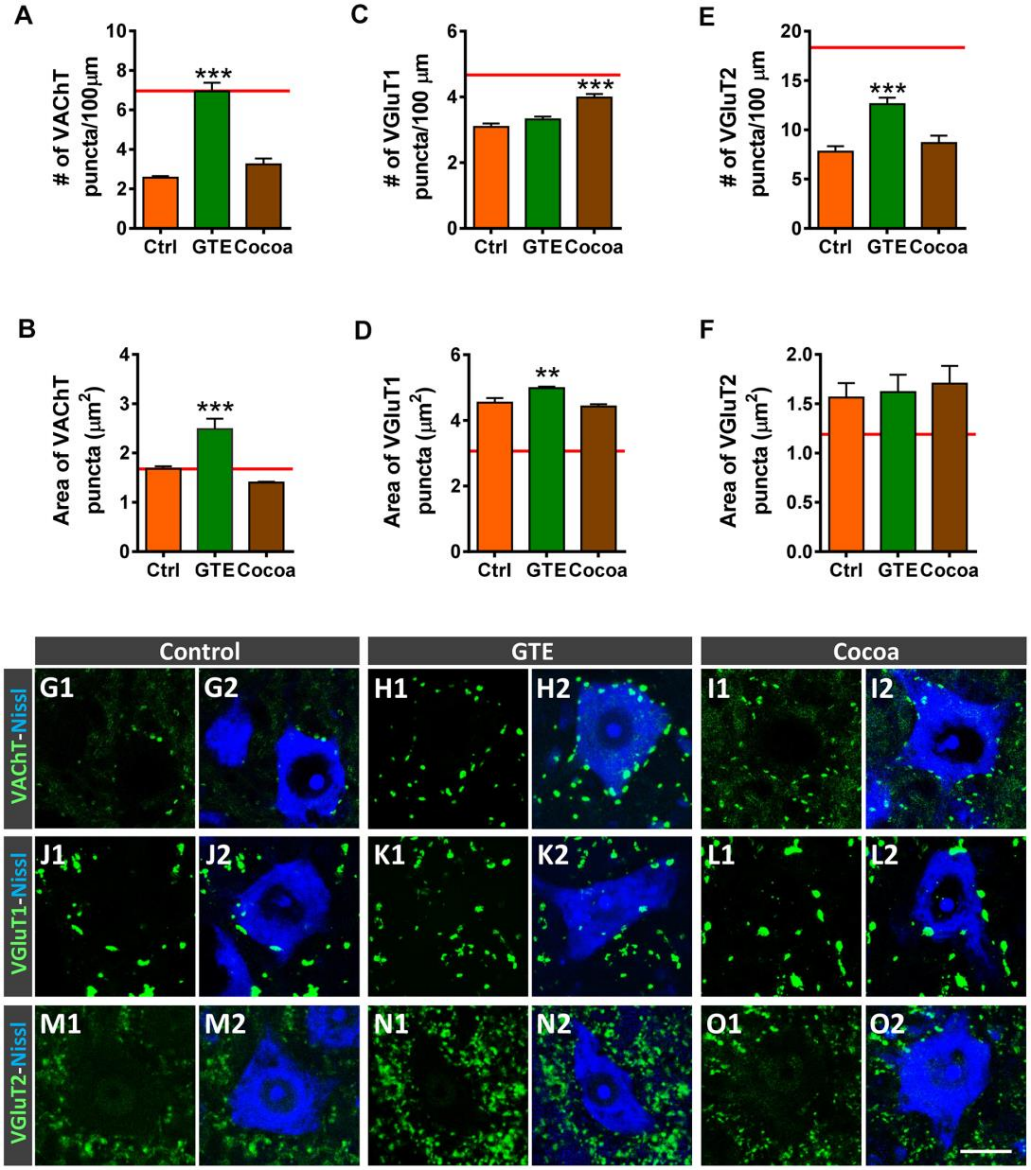
**Effects of GTE- and cocoa-supplemented diets on excitatory cholinergic (VAChT-positive) and glutamatergic (VGluT1 and VGluT2) synaptic inputs to aged spinal MNs.** (**A**–**F**) Graphs show the average density (number of puncta per 100 μm of MN soma perimeter, **A**, **C**, **E**) and size (in μm^2^, **B**, **D**, **F**) of the different types of afferent synapses examined; the red dashed line in each graph indicates the mean value of the corresponding afferent synapse density or size found in adult mice [6]. (**G1**–**O2**) Representative confocal micrographs of VAChT, VGluT1 and VGluT2 nerve terminals contacting MN cell bodies of animals from control, GTE and cocoa groups, as indicated. Spinal cord sections were immunolabeled for either VAChT, VGluT1 or VGluT2 (green), and counterstained with fluorescent Nissl staining (blue) to visualize MN cell bodies, as indicated in panels. Data in the graphs are expressed as the mean ± SEM, **p < 0.01 and ***p < 0.001 vs. control (one-way ANOVA, Bonferroni’s *post hoc* test); 50-60 MNs were analyzed per animal (number of animals per group: control =3, GTE = 4; and cocoa = 5). Scale bar in **O2** = 20 μm (valid for **G1**–**O1**).

We and others have reported that aging is accompanied by a significant depletion of afferent inputs to MNs [6, 76, 77]. Thus, with age, MNs suffer a loss of excitatory cholinergic and glutamatergic inputs, with no overt changes in the density of inhibitory GABAergic afferent synapses. We examined then whether supplementations with GTE or cocoa flavanols were able to prevent the process of aging-associated MN deafferentation. Spinal cord sections from old mice were immunostained with antibodies against VAChT, vesicular glutamate transporter 1 (VGluT1) and vesicular glutamate transporter 2 (VGluT2) (for cholinergic [C-boutons] and glutamatergic excitatory synapses, respectively), and the number and size of labeled synaptic puncta on MN somata were analyzed. We found that, in comparison with MNs of control mice, those of animals that received GTE-, but not cocoa-, supplementation exhibited significantly higher density and larger area of cholinergic synaptic terminals (Figure 5A, 5B, 5G1–5I2), and significantly increased density of VGluT2-positive synapses (Figure 5E, 5F, 5M1–5O2). Conversely, a significant increase in the density of VGluT1-positive synapses was observed on MNs of cocoa group animals; MNs of GTE group mice, however, exhibited no significant changes in the number of VGluT1-contacting boutons with respect to those on control MNs, although VGluT1 synapses on the GTE group MNs showed a significant increased size (Figure 5C, 5D, 5J1–5L2).

**Figure 6:**
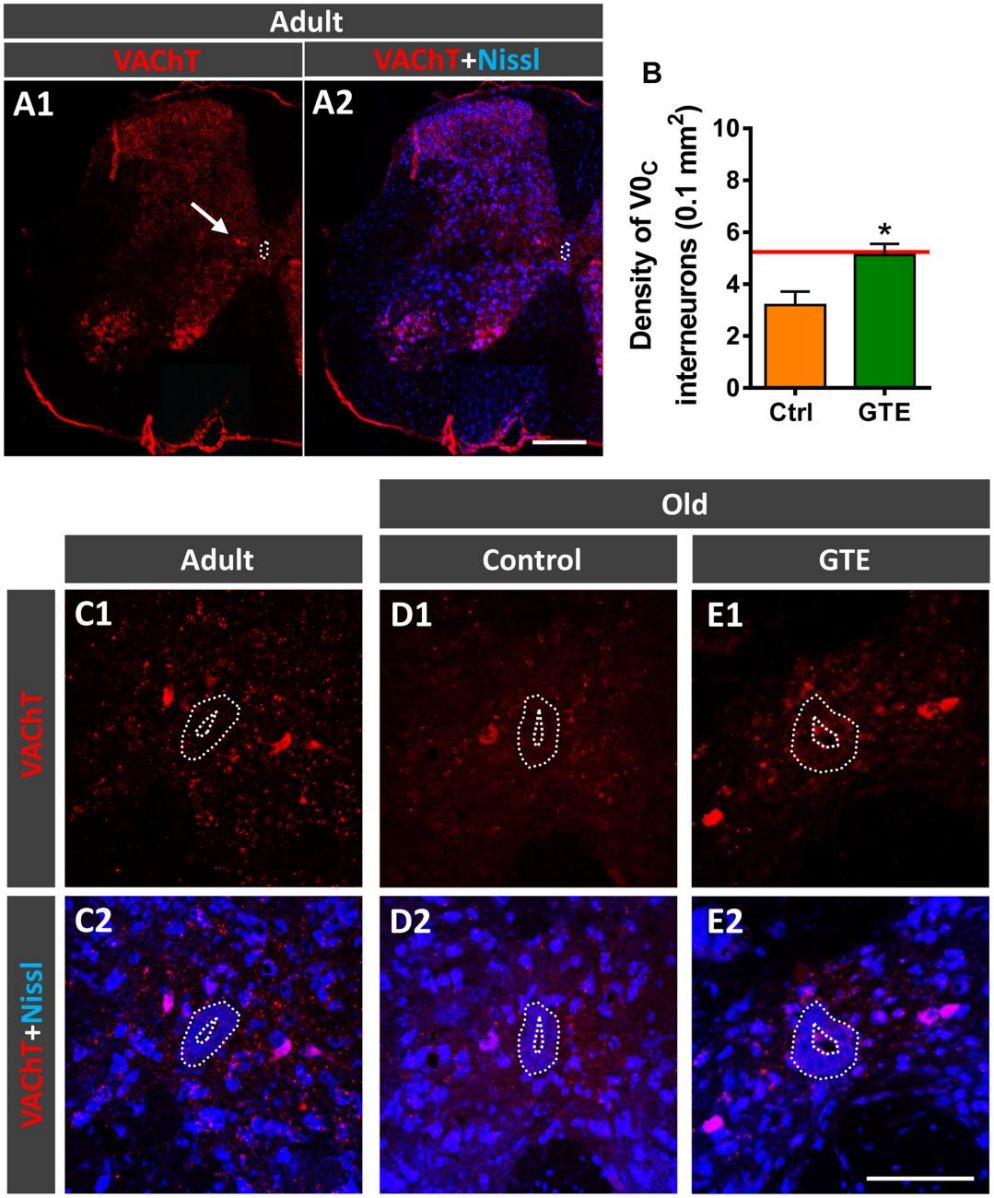
**GTE-supplemented diet prevents the age-related loss of V0_C_ interneurons.** (**A1**, **A2**) A general view of a spinal cord hemisection of an adult mouse immunolabeled for VAChT (red) and counterstained with fluorescent Nissl (blue) for neuron identification; the arrow points to a V0_C_ interneuron cluster located near the central canal (delimited by a dotted line); note also the different VAChT-positive MN pools in the ventral horn. (**B**) Density of V0_C_ interneurons in spinal cords of aged animals from control and GTE groups; bars represent the mean ± SEM of 3-5 animals (20-28 images) per condition; *p < 0.05 vs. control (Student’s t-test); the red line indicates the mean value in adult mice found in [6]. (**C1**–**F2**) Representative confocal micrographs of VAChT-positive V0_C_ interneurons (red) in the spinal cords of an adult mouse (**C1**, **C2**) and of old animals fed with the control (**D1**, **D2**) and GTE-supplemented diet (**E1**, **E2**); sections were counterstained with fluorescent Nissl (blue) for neuron visualization; the central canal is delimited by dotted lines. Scale bars: in **A2** = 200 μm (valid for **A1**), and in **E2** = 100 μm (valid for **C1**–**E1**).

Cholinergic C-bouton, VAChT-positive, inputs to MNs arise from small clusters of cholinergic interneurons which express the paired transcription factor *Pitx2* and are distributed in a longitudinal column near the central canal of lumbar spinal cord [78]. Due to their cholinergic phenotype, these interneurons, referred to as V0_C_ interneurons, can be labeled with antibodies against choline acetyltransferase (ChAT) or VAChT (Figure 6A1, 6A2). In lumbar spinal cord sections of adult and old mice we counted the number of VAChT-immunolabeled V0_C_ interneurons in an area of 0.1 mm^2^ around the central canal. In comparison with adult mice, aged animals exhibited a prominent tendency to reduce (~30%) the density of V0_C_ interneurons (adult: 5.2 ± 0.4, old: 3.6 ± 0.6 [mean ± SEM]; n = 4 animals per group, *p* = 0.07, Student’s t-test). The decline in the number of V0_C_ interneurons found with aging correlates with the reduction in C-bouton density observed on aged MNs, suggesting that cholinergic deafferentation of MNs is, at least in part, due to the loss of V0_C_ interneurons. Since GTE prevented the loss of C-boutons on MNs, we examined whether this supplement was able to prevent the aging-associated decline of V0_C_ cells and found that mice fed with GTE-enriched diet exhibited significant higher density of such interneuron population compared to control animals (Figure 6B–6E2). Thus, the beneficial action of GTE on C-bouton synapse stabilization is in concordance with its capacity to preclude V0_C_ interneuron loss.

### Impact of dietary supplementations with GTE and cocoa flavanols on age-related gliosis in ventral horn spinal cord of old mice

**Figure 7:**
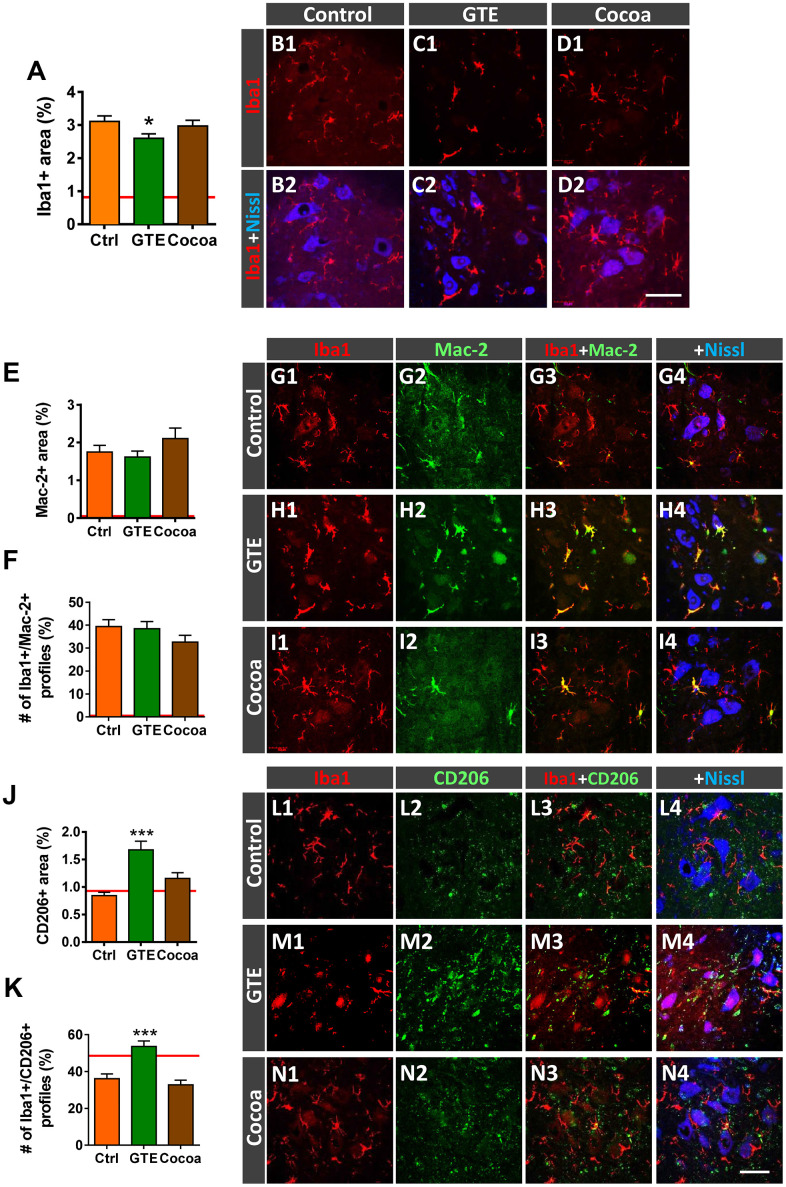
**Impact of GTE- and cocoa-supplemented diets on the aging-associated microgliosis and imbalance in M1/M2 microglial phenotypes found in the ventral horn spinal cord of old mice.** Lumbar spinal cord sections were immunostained for the microglial marker Iba1 (red), and either Mac-2 or CD206 (green), for M1 or M2 microglia, respectively; fluorescent Nissl staining (blue) was used for MN visualization. (**A**) Quantification of microglia expressed as the percentage of ventral horn occupied by Iba1-positive profiles. (**B1**–**D2**) Representative confocal images showing Iba1-staining around spinal cord MNs of animals from control, GTE and cocoa groups as indicated in panels. (**E**, **F**, **J**, **K**) Quantification of Mac-2-positive (**E**) and CD206-positive (**J**) profiles surrounding MNs shown as the percentage of ventral horn area occupied by the immunostained profiles; the proportion of microglial profiles expressing both Iba1 and either Mac-2 (**F**) or CD206 (**K**) is also shown. The average values of these parameters in adult mice from our previous study [6] are indicated in each graph (red lines) for comparison purposes. (**G1**–**I4**, **L1**–**N4**) Representative confocal micrographs used for data analysis showing Mac-2 (**G1**–**I4**) and CD206 (**L1**–**N4**) in combination with Iba1 and fluorescent Nissl staining, as indicated in panels. Data in the graphs are expressed as the mean ± SEM; a total of 45-50 images per experimental group were analyzed (number of animals per group: control [Ctrl] = 3, GTE = 4, cocoa = 5); *p < 0.05 and ***p < 0.001 vs. Ctrl (one-way ANOVA, Bonferroni's *post hoc* test). Scale bar in **N4** = 50 μm (valid for **B1**–**D1**, **G1**–**I4**, **L1**–**N3**).

We have previously reported that, in parallel with central synapse loss, a prominent gliosis occurs in the spinal cord ventral horn of old mice, suggestive that aging is accompanied by a neuroinflammatory response around MNs [6]. Glia, particularly microglia, appear to play a major role in synapse elimination during development and in pathological conditions; moreover, glia can indirectly alter synaptic function throughout the release of pro-inflammatory mediators (recently reviewed by [79]). We wanted to examine whether a diet supplemented with either GTE or cocoa flavanols is able to prevent aging-associated microgliosis in spinal cord. Ionized calcium-binding adaptor molecule 1 (Iba1)-immunoreactivity, used as a specific marker for microglia, was analyzed in ventral horn. Compared to control mice, a moderate, although significant, reduction in Iba1-immunolabeling was noticed in spinal cords of GTE group mice, but not in those of cocoa group (Figure 7A, 7B1–7D2). Aging-associated spinal cord neuroinflammation is accompanied by structural changes in microglial cells, which exhibit increased morphological complexity and branching degree [6]. By using the skeleton approach [80], we analyzed and compare microglia morphology in aged animals of the three experimental groups and found no differences between them. Thus, our flavonoid-enriched diets do not to promote any overt changes in microglial complexity (Supplementary Figure 5A, 5F3).

**Figure 8:**
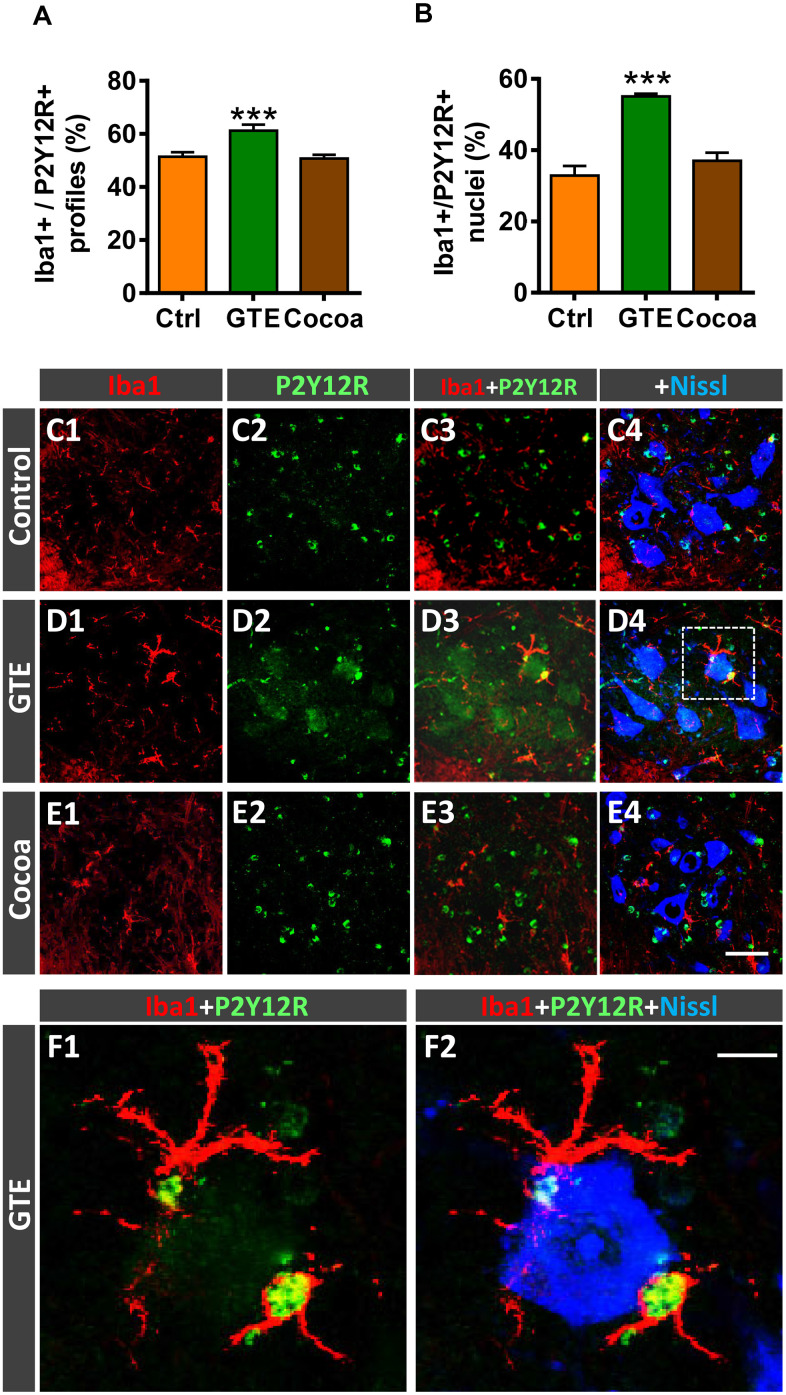
**Impact of GTE- and cocoa-supplemented diets on P2Y12R expression in spinal cord microglia of old mice.** Sections of lumbar spinal cords from mice of different experimental groups were double immunostained for Iba1 and P2Y12R. (**A**, **B**) Quantification of Iba1-positive profiles also exhibiting P2Y12R immunoreactivity (**A**) and of those displaying nuclear P2Y12R expression (**B**). (**C1**–**F2**) Representative confocal micrographs used for data analysis showing P2Y12R (green) in combination with Iba1 (red) and fluorescent Nissl staining (blue, for MN visualization), as indicated in panels. A higher magnification of area delimited by the dashed square in **D4** is shown in (**F1**, **F2**). Note the nuclear expression of P2Y12R in Iba1-positive microglial cells in close contact with a MN. Data in the graphs are expressed as the mean ± SEM; a total of 40-50 images per experimental group were analyzed (number of animals per group: control [Ctrl] = 3, GTE = 4, cocoa = 5); ***p < 0.001 vs. Ctrl (one-way ANOVA, Bonferroni's *post hoc* test). Scale bar: 50 μm in (**E4**) (valid for **C1**–**E3**) and 10 μm in (**F2**) (valid for **F1**).

Microglia are plastic cells which can be activated to produce detrimental (cytotoxic) or beneficial (neuroprotective) effects on neurons [81, 82]. Thus, microglia can be categorized into two opposite phenotypes M1 and M2; whereas M1 are proinflammatory cell types, M2 play a role as downregulators of inflammation. We have shown that age-related reactive microgliosis in spinal cord entails an imbalance in M1/M2 phenotypes, which leads to a polarization to harmful activated M1 state [6]. To examine whether the supplemented diets tested here were able to modulate this microglial phenotype activation, Iba1 immunohistochemistry was combined with either Mac-2 or macrophage mannose receptor (CD206) immunolabeling for M1 or M2 microglia, respectively [82–84]. No overt changes were found in Mac-2-immunolabeled (M1) microglia in GTE and cocoa groups when compared with control group (Figure 7E–7I4). However, in comparison with the latter, we found that spinal cords of the GTE group exhibited a significant higher density of beneficial CD206-positive, M2, microglia around spinal MNs; this change was not found in animals that received a dietary supplementation with cocoa flavanols (Figure 7J–7N4). To further study the impact of our flavonoid-supplemented diets on modulating microglia, we examine the expression of the G-protein-coupled purinergic receptor P2Y12R in ventral horn. P2Y12R is exclusively expressed in microglia and allows to distinguish these cells from infiltrating monocytes and other peripheral macrophages [85–87]. P2Y12R has been shown to be increased during anti-inflammatory conditions and is considered as a marker for M2-like microglia [88, 89]. Spinal cord sections were double immunolabeled for Iba1 and P2Y12R, and the number and density of Iba1-positive profiles exhibiting P2Y12R positivity were analyzed in ventral horn. Compared to control spinal cords, those of animals fed with the GTE-, but not cocoa-, supplemented diet displayed a significantly higher amount of Iba1-positive profiles also expressing P2Y12R (Figure 8A, 8C1–8E4). Additionally, we noticed that whereas in the vast majority of control microglial profiles P2Y12R exhibited a cytoplasmic expression, in those of GTE group, P2Y12R-immunoreactivity is mainly located in the nucleus (Figure 8B, 8F1, 8F2), as previously reported to occur for M2 phenotype [90]. Overall, these findings indicate that age-associated reactive microgliosis can be potentially modulated to M2 neuroprotective phenotype by GTE.

**Figure 9:**
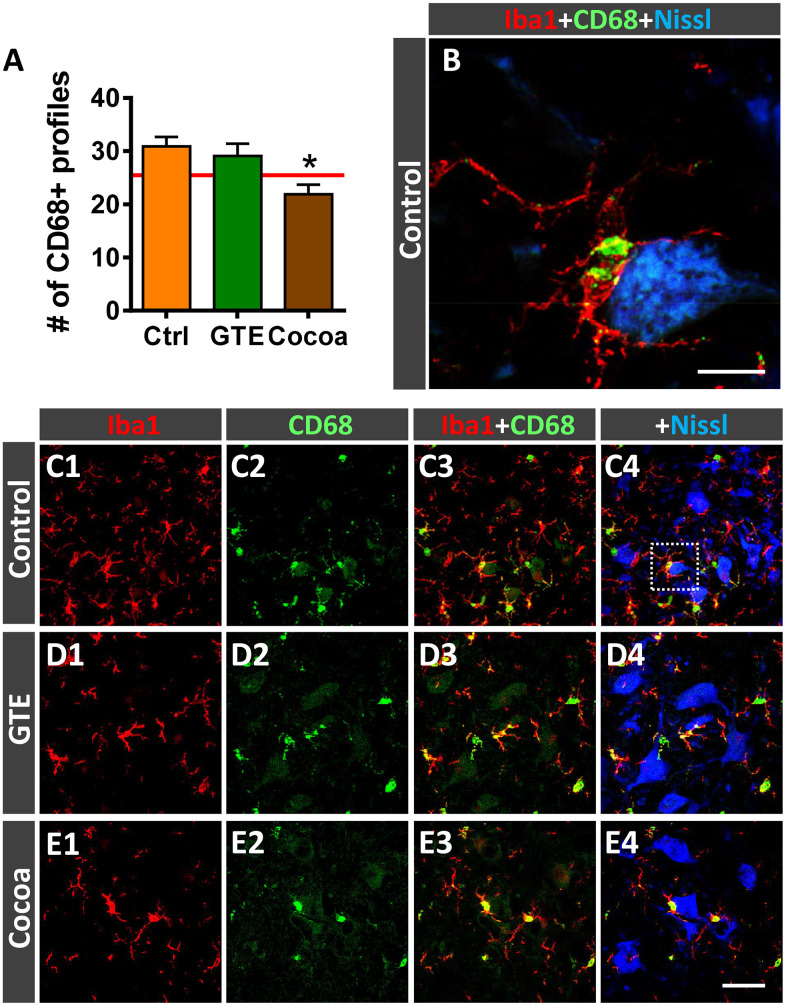
**Impact of GTE- and cocoa-supplemented diets on microglial activation in ventral horn spinal cord of old mice.** Sections of lumbar spinal cords from mice of different experimental groups were double immunostained for Iba1 and CD68, a marker of activated phagocytic microglia. (**A**) Quantification of CD68-positive profiles around MNs in control, GTE and cocoa groups. (**B**–**E4**) Representative confocal micrographs used for data analysis showing CD68 (green) in combination with Iba1 (red) and fluorescent Nissl staining (blue, for MN visualization), as indicated in panels. A higher magnification of area delimited by the dashed square in **C4** is shown in (**B**). Data in the graph are expressed as the mean ± SEM; a total of 40-50 images per experimental group were analyzed (number of animals per group: control [Ctrl] = 3, GTE = 4, cocoa = 5). *p < 0.05 vs. Ctrl (one-way ANOVA, Bonferroni's *post hoc* test). Scale bar: 10 μm in (**C**) and 50 μm in (**E4**) (valid for **C1**–**E3**).

Activated microglia undergo changes in the expression of the cluster differentiation 68 (CD68) protein, a member of the lysosomal/endosomal-associated membrane glycoprotein (LAMP) that regulates phagocytosis in macrophage lineage cells. CD68 is present in lysosomes and is commonly considered a marker of activated phagocytic microglia [91]. Resting mouse microglia exhibit some basal expression of CD68, which is prominently upregulated after activation [92]. Double immunohistochemistry for CD68 and Iba1 in spinal cords of adult and old mice revealed that virtually all CD68-positive profiles are located inside Iba1-immunolabeled cells. Interestingly, compared with adult spinal cords, those of aged mice exhibited a significant increase in CD68-expression in microglial cells located around MNs, as revealed by the percentage of ventral horn area occupied by CD68-positive profiles (adult mice: 3.12 ± 0.63 %, n = 4; old mice: 18.85 ± 1.58 %, n = 5; *p* < 0.0001, Student’s t-test). Moreover, individual Iba1-immunolabeled microglial cells of aged mice exhibited significant larger CD68-positive puncta than those of adult animals (area of CD-68-positive profiles: adult mice, 2.85 ± 0.24 μm^2^, n = 4; old mice, 10.71 ± 0.69 μm^2^, n = 5; *p* < 0.0001, Student’s *t*-test; 60 CD68-stained profiles per animal were analyzed). These data indicate that microgliosis observed in the spinal cord in the course of aging is associated with a prominent increase in activated phagocytic microglial cells. Whereas no changes in the density of CD68-positive microglial cells were found in ventral horns of GTE-supplemented aged mice, the number of those profiles surrounding MNs was found significantly reduced in animals from cocoa group compared to controls (Figure 9A–9E4). This indicates that cocoa-, but not GTE-, flavonoids are able to prevent the activation of microglia and their increased phagocytic activity in the course of aging.

### Changes in age-related alterations of DRG sensory neurons induced by either GTE- or cocoa-supplementation intake

We have reported that aging is associated with changes in nociceptive (CGRP-positive and *Bandeiraea simplicifolia* lectin [isolectin B_4_, IB4]-positive) and proprioceptive (parvalbumin [PV]-positive) DRG neurons [6]. Thus, we have shown that L4 DRGs of aged mice exhibited increased proportions of sensory neurons expressing CGRP and PV, and stained with IB4. These changes have been found to be accompanied by a marked atrophy of cell bodies of all those neuronal populations. Here, we analyzed the effects of GTE and cocoa-flavanols on these age-related sensory neuron alterations. We found that, compared to control group, DRGs of GTE- or cocoa-supplemented animals displayed a significant decrease in the number of CGRP-positive and IB4-positive neurons; additionally, cocoa, but not GTE, promoted a significant enlargement of soma size of those neuronal populations. Moreover, cocoa, but not GTE intake, also significantly reduced the number, although increased soma size of PV-positive neurons (Supplementary Figure 6A–6O2).

### GTE and cocoa flavanols do not impact locomotor activity decline or electromyographical changes found to occur with aging

The effects of GTE or cocoa supplementation on aging-related motor decline were examined using open-field and pen tests. In a previous study [6], we have shown that aging results in a gradual reduction in the locomotor activity, as measured after the automated recording of mouse movements using the open-field test. We found that neither GTE nor cocoa supplementation improved the impaired motor performance of old mice assessed with this test; in fact, motor activity, measured by open-field test, was even noticed to be slightly (although not significantly) reduced at the end of the experimental period in animals fed with any of the two tested supplemented diets (Supplementary Figure 7A, 7B). Additionally, compared to control diet, neither GTE nor cocoa supplementation modified the parameters of pen test performance observed in old mice (not shown).

We have previously demonstrated that aging in mice is accompanied by noticeable electrophysiological changes in form of significant reduction in nerve conduction velocity and compound muscle action potential (CMAP) amplitude and, also, by a mild decrease in functional motor unit number [6]. We examined whether GTE or cocoa supplemented diets were able to improve the age-related alterations in such electrophysiological parameters. Neither green tea extract nor cocoa intake induced any significant change in either the CMAP latency or CMAP amplitude found in old animals (Supplementary Table 1). The excitability of the H reflex circuit was not significantly different, although the cocoa group had a lower H/M amplitude ratio closer to normal young adult values. It is of interest to note that the motor unit number estimation (MUNE) test gave a slight increase in the number of motor units in the TA muscle of both supplement groups compared with the control old group, which is in agreement with the results of NMJ innervation detailed above.

## DISCUSSION

To evaluate nutritional ingredients that can mitigate sarcopenia progression, we tested the effects of two-flavonoid enriched plant extracts, green tea catechins or cocoa flavanols. Besides their potential benefits on aged muscles, the impact of these dietary supplements on aging-associated alterations in other constituents of the neuromuscular system was also examined. Our results indicate that GTE- and cocoa-flavonoid intake may ameliorate some aging-related neuromuscular regressive phenomena occurring in mice by: *a)* preventing MN deafferentation; *b)* elevating the proportion of anti-inflammatory microglial phenotypes, found to be reduced in the aged spinal cord; *c)* improving the degree of muscle innervation and NMJ maturity; and *d)* delaying some potential harmful aging-related changes in skeletal muscles.

Although GTE or cocoa supplementations are able to increase the survival rate of mice, we did not notice a significant impact on the motor activity decline or on the explored electrophysiological alterations associated with senescence. These results contrast with those from previous studies showing benefits of GTE catechins or cocoa flavanols on physical activity and muscle function in rat and mouse models of muscle dysfunction. In aged rats, GTE has been reported to improve muscle function after reloading following disuse [36]. In a murine model of accelerating aging, GTE catechins along with habitual exercise have been shown to suppress the aging-related decline of physical performance [34]. Other studies have revealed that GTE and its major catechin EGCg improve muscle function and endurance capacity in mouse models of Duchenne muscular dystrophy [30, 31]. The disparity between these and our results could be explained by differences in the animal models, and tests used to evaluate motor behavior and muscle function. Moreover, it is reasonable to assume that the tests used in the present study to assess motor phenotype of mice, although allow us to detect an age-related decline in muscle function [6] are not sensitive enough to detect subtle improvements in motor activity promoted by the dietary intervention. On the other hand, a previous study has shown that the cocoa flavanol-epicatechin could improve the age-associated decline of physical activity in mice [46]. However, this intervention was carried out for a longer length of time (~ 9 months) than our study, and the dose of epicatechin is much higher than that delivered in the present work. It is possible that higher doses of cocoa flavanols and longer intervention periods may be needed to impact motor output. Nevertheless, our study identified positive structural changes in the neuromuscular system as well as in muscle regenerative and energy pathways, a harbinger of positive impact of our tested compounds on the muscle.

### Effects of green tea- and cocoa-enriched diets in skeletal muscles of aged mice

In the present study, we did not observe an effect of GTE or cocoa flavanol supplementations on mass, fiber size and density, or connective tissue content of aged muscles. Nevertheless, both dietary supplements significantly: *a)* prevented the age-associated decline of SC numbers [6, 93, 94]; *b)* reduced the density of fibers with central nuclei (taken as an indicator of a degenerative/regenerative process of muscles [6, 11, 49, 95]); and *c)* attenuated the accumulation of the age pigment lipofuscin in myofibers [6]. SCs are essential for skeletal muscle regeneration [96–98], and the age-associated decline in number and function of SCs negatively affects the capacity to replace and/or repair damaged myofibers (reviewed by [53, 99]). However, although SCs are crucial for the genesis of new fibers during the regenerative process of the aged muscle, they do not seem to have any impact on fiber size during aging [100]. This is in agreement with the absence of significant changes in the cross-sectional area of myofibers we found in flavonoid-supplemented animals, despite the increased proportion of SCs noticed in muscles. Previous studies have shown that caloric restriction is able to preclude the age-related loss of myofibers [11] and to enhance SC function and muscle repair [20]. This indicates that metabolic factors are critical in the regulation of myofiber turnover and myogenic activity of SCs. The beneficial effects of caloric restriction on aged muscles appear to be associated to the enhancement of mitochondrial biogenesis and oxygen consumption resulting from this dietary intervention [20]. On the other hand, EGCg has been reported to significantly promote SC proliferation and differentiation in muscles of senescent rats after muscle disuse recovery [35, 36]. This action, which appears to be mainly mediated by oxidative stress inhibition, is able to attenuate the muscle mass loss but fails to improve muscle recovery during reloading following inactivity [36]. EGCg has also been shown to promote SC activation in aged mice and humans, although this effect has been mainly ascribed to the reduction of myostatin levels induced by the compound [101]. Here, we confirm the efficacy of GTE in maintaining the SC population in aged mice and also provide evidence that cocoa flavanols have a similar impact on preventing age-related SC loss. The benefits of these flavonoids on aged muscles could be a consequence of the enhancement and acceleration of myofiber turnover and repair, which would be facilitated by the increased SC proliferation, as reported previously for green tea catechins [99]. Nevertheless, the possibility that our dietary supplements promote a beneficial action by slowing down the aging process of muscles and, in this way, diminishing the number of SCs used for muscle regeneration, cannot be excluded. Indeed, aged muscles of animals that received the flavonoids, exhibited a significant increase in the proportion of apparent younger myofibers, with reduced lipofuscin deposition and decreased frequency of centrally located nuclei.

Due to the reported antioxidant activities of flavonoids [22, 43, 102], it is tempting to ascribe the muscular benefits of GTE and cocoa flavanols to their protective effects against reactive oxygen species. Reactive oxygen species are by-products of mitochondrial oxidative phosphorylation, which are increased in aging muscles due to mitochondrial dysfunction [60]. On the other hand, mitochondria play a crucial role in the regulation of apoptosis in the senescent muscle [103, 104]. Thus, age-related mitochondrial dysregulation appears to be a critical factor contributing to muscle wasting in the elderly [105, 106]. PGC-1α is considered an essential transcription factor in the mitochondrial biogenesis and function [57], and its overexpression has been reported to increase mitochondrial mass [107]. Additionally, PGC-1α is also critical in regulating numerous genes involved in muscle morphology and function throughout its interactions with signaling pathways that control mitochondria homeostasis and protein balance [108]. PGC-1α content and activity have been shown to be reduced in aged muscles, although these findings are not free from certain controversy (reviewed by [108]). Redox regulation is an important mechanism in controlling PGC-1α activity. Indeed, reactive oxygen species could negatively regulate PGC-1α in aged skeletal muscle, as reported in cardiac myocytes [109]. On the other hand, there is evidence that PGC-1α is able to activate the expression of endogenous antioxidant proteins (including superoxide dismutase and glutathione peroxidase), exert anti-inflammatory effects, promote neovascularization, and protect the senescent muscle from increased degradative processes such as proteolysis, autophagy and apoptosis [110–112]. Chronic physical training increases the levels of PGC-1α in the skeletal muscle [113–115]. Here, we found that animals fed with either GTE- or cocoa flavanols exhibited increased muscular content of PGC-1α compared to controls. The augmented levels of PGC-1α after either of the two diets tested were observed in both TA (fast) and Sol (slow) muscles, although the effects on this transcription factor expression were slightly more prominent with GTE than with cocoa supplementation. In fact, we did not find statistically significant changes in the muscular content of PGC-1α between control and cocoa group, although latter exhibited a remarkable increased expression of the transcription factor in both TA and Sol muscles. As aforementioned, reductions in PGC-1α expression impairs mitochondrial dynamics, structure and function in muscles [105]. Although a number of metabolic regulators besides PGC-1α could be involved in the maintenance of mitochondrial homeostasis, our results suggest that the beneficial effect of GTE and cocoa supplementation on mitochondrial content in aged muscles could be due, at least in part, to their capacity of restoring PGC-1α levels.

### Effects of green tea- and cocoa-enriched diets in neuromuscular junctions of aged mice

Aging is accompanied by structural and molecular changes at NMJs (reviewed by [5]). Although it is not yet clear whether these are a consequence of progressive aged-related alterations in either MNs or muscle fibers, it has been unambiguously demonstrated that an important proportion of NMJs in murine models of aging exhibit signs of partial or complete denervation, and changes compatible with structural remodeling [6, 9, 11, 63–65]. Here we found that some alterations in aged NMJs of mice could be mitigated by any of the two flavonoids-enriched diets tested. Indeed, compared to controls, muscles from animals fed with either GTE- or cocoa flavanol-supplements displayed a significant reduction in the proportion of denervated or partially innervated NMJs. Moreover, we also noticed that muscles of GTE and cocoa groups showed a significant decline in the number of fragmented endplates, and increased amounts of them exhibiting a pretzel-like appearance, indicative of a higher degree of NMJ maturity. Nevertheless, it is necessary to take into account that, compared to mouse NMJs, those from humans are structurally more stable across the entire adult lifespan, showing scarce signs of degeneration or remodeling with age [116]. This aspect must be taken into consideration regarding the applicability to our results to humans.

These improvements promoted by the dietary supplements in age-related NMJ alterations were not accompanied by significant changes in the expression of CGRP, GAP-43, or agrin, three developmentally regulated molecules involved in NMJ development, maintenance and plasticity. All them are synthetized in MN cell bodies, axonally transported and released at nerve terminals. CGRP is a neuropeptide which is highly expressed in NMJs during development, decreases its content with synaptic maturation, but is upregulated during nerve sprouting and muscle reinnervation [66–69]. Agrin, throughout the clustering of AChRs, plays an essential role in the initiation of NMJ formation, and later in the maintenance and regeneration of adult NMJs [70]. GAP-43 is expressed in growth cones, and its content is increased during motor axonal growth and plasticity, and in regeneration following axonal injury [71]. We have previously reported that the content of these synaptic proteins decreases in the course of the postnatal development, with NMJs of adult mice exhibiting significantly lower protein levels than those of young animals; nevertheless, their expression increases again in aged NMJs, suggesting a role for these molecules in the promotion of compensatory nerve sprouting and reinnervation of denervated muscles in aging [6]. We observed that, GTE- and cocoa flavanols only promoted minor increases in the levels of CGRP and agrin regularly found in aged NMJs. Additionally, no significant differences were observed in GAP-43 expression at the NMJs of flavonoid-supplemented animals, although moderate reductions of this protein were noticed when determined by western blot in the total muscle. Overall, these results indicate that, at least at the concentrations and dietary regimens used here, our supplements are not able to significantly modulate the expression of these proteins linked to NMJ maintenance. The modest decline in GAP-43 content found in muscles of GTE and cocoa groups correlates in some way with the reduction of nerve terminal sprouting observed in NMJs following dietary supplementations and suggest that, in those conditions in which aged NMJs are more stable, intramuscular nerves have a diminished need to sprout for maintaining proper levels of innervation.

In our knowledge, this is the first study reporting the attenuation of age-associated alterations in NMJs promoted by flavonoid-enriched diets.

### Effects of green tea- and cocoa-enriched diets in preserving afferent synaptic inputs to motoneurons

An interesting finding of our study is the prevention of age-associated MN deafferentation [6, 76, 77] observed, particularly, following GTE intake. As we have previously reported, aging is accompanied by a prominent loss of excitatory cholinergic (C-boutons) and glutamatergic synapses on MNs [6], a pathological change also observed in different murine models of MN diseases, such as spinal muscular atrophy (SMA) [117–121] and amyotrophic lateral sclerosis (ALS) [117, 122–125], and after nerve injury [126–128]. It is well known that MN excitability is accurately regulated by afferent synaptic inputs which mediate the genesis of coordinated firing patterns resulting in the appropriate muscle contraction during motor activities [129]. Since the control of MN excitability appears to be crucial for motor function, it seems clear that age-associated MN deafferentation can markedly alter the normal electrophysiological patterns and impair motor skills of aged mice, as we previously reported [6]. In this regard, cholinergic C-bouton synapses appear to be particularly important in the control of motor activity during locomotion, being involved in the task dependent regulation of motor outputs [130]. C-boutons also determine the existing differences in intrinsic cellular excitability between MN subtypes innervating slow- or fast-twitch muscles [131, 132]. Thus, MNs innervating slow-twitch muscles (slow MNs) have a higher excitability and slower firing than MNs innervating fast-twitch muscles (fast-fatigable MNs), and it is that degree of intrinsic excitability which confers to slow MNs a higher resistance to degeneration compared to fast fatigable ones. Our results indicate a differential effect of GTE and cocoa supplementation in the preservation of excitatory synaptic afferents on aged MNs. GTE-, but not cocoa-, flavonoids promoted protection against cholinergic C-bouton- and glutamatergic VGluT2 synapse-deafferentation. However cocoa flavanols, but not GTE, reduced the age-associated decline of glutamatergic VGluT1 synaptic puncta on MNs. These results suggest the need to combine distinct flavonoids in order to achieve a wider effectiveness on MN afferent preservation during aging.

In an attempt to explore further the cellular targets of these flavonoids linked to their preventive benefits on age-associated MN deafferentation, we examined potential changes in the neuronal sources of C-boutons, VGluT1 and VGluT2 synapses contacting spinal MNs. GTE significantly increased the number of V0_C_ interneurons (the source of motoneuronal C-boutons), which were depleted in control aged spinal cords. This suggests that the attenuation of aging-related C-bouton loss is, in part, a consequence of the flavonoid action on the maintenance of V0_C_ interneurons during senescence. On the other hand, a substantial number of VGluT1 synaptic terminals on MNs arise from DRG proprioceptive (PV-positive) sensory neurons that peripherally innervate muscle spindles, stretch-sensitive mechanoreceptors embedded in skeletal muscles. These VGluT1 proprioceptive terminals, named Ia afferents, establish highly selective excitatory monosynaptic connections with spinal MNs, and are essential for the muscle stretch reflex and, consequently for posture and motor coordination [133]. Our finding that cocoa, but not GTE, are able to remarkably ameliorate aging-related changes found in proprioceptive PV-positive DRG neurons correlates with the effects of this flavonoid on preventing VGluT1 afferent synaptic loss on aged MNs. This suggests that the improvement provided by cocoa flavanols on aging-associated glutamatergic MN deafferentation could be mediated, at least in part, by their actions on DRG sensory neurons.

Despite the preventive action of these flavonoids on age-related MN deafferentation, this effect was not associated to any noticeable improvement in motor axon degeneration that occurs with age [6, 72]. This could contribute to explain the lack of improvement of the electromyographical alterations observed in aged animals [6]. Our results also suggest that preventing MN deafferentiation is not enough to achieve the appropriate morphofunctional state of MNs necessary for maintaining or ameliorating the impaired motor activity. On the other hand, more studies are required to examine whether the preserved synaptic afferents are able to mediate a proper neuromodulatory action for an accurate MN firing and adjust muscle activation to demands of different motor tasks.

### Effects of green tea- and cocoa-enriched diets in preventing age-associated microgliosis

Interestingly, GTE supplementation induced conspicuous changes in aging-associated spinal cord microgliosis. We have previously shown that age is accompanied by a neuroinflammatory reaction in ventral horn, which is particularly prominent around MNs [6]. Neuroinflammation in aged spinal cord could be considered as a part of a more general process chronically affecting different regions of the central nervous system, as reported in healthy elderly [134, 135]. The fact that neuroinflammation has been found to be also associated to a number of neurodegenerative disorders, including the MN diseases ALS and SMA [118–120, 136–139], suggests that age-related gliosis can enhance the neuronal vulnerability to dysfunction and degeneration. Gliosis in aged spinal cord entails a marked increase in harmful microglial (M1) and astroglial (A1) phenotypes, and a depletion of neuroprotective M2 microglia and A2 astroglia [6]. Activation of proinflammatory M1 and A1 cells in spinal cord during aging could exert detrimental actions on MNs that may negatively affect their structure and function, with the subsequent impairment of NMJs. In this regard, it has been recently shown that pharmacological reduction of microglia is able to maintain the NMJ integrity during normal aging [140]. This indicates that microglial activation is a critical step in the pathogenic changes leading to neuromuscular decline during aging. However, the cellular mechanisms by which microglia exert their detrimental actions in the aged neuromuscular system, and how we can modulate the microglial reaction in aging are not clarified. A line of evidence assigns to microglia an active role in synapse elimination during neurodegeneration, as occurs in ALS, and after peripheral nerve injury [79, 128]. Therefore, age-related changes in the microglial cell state could be responsible for the synaptic loss observed on aged MNs. Thus, it is tempting to assume that aging entails an altered microenvironment in the spinal cord, which triggers a microglial reaction leading to central synapse elimination. Indeed, in a model of sciatic nerve axotomy, we have recently demonstrated that microglia are recruited by injured MNs and displayed positive chemotaxis to afferent synapses; activated microglia stablish close contacts with degenerating afferent terminals, suggesting a role for those cells in the disintegration of defective synapses on MNs [128]. We do not know, however, whether microglial activation in aging is a primary event resulting in the elimination of normal functional nerve afferents on MNs with the consequent neuronal impairment, or a secondary process occurring in response to a previous altered synaptic function. Nevertheless, as we show here, aging-associated imbalance in M1/M2 phenotypes may be partially modulated by a dietary intervention based on GTE. In fact, we found that a diet enriched with GTE, but not cocoa flavanols, not only significantly reduces the density of microglial profiles, but also increases the proportion of neuroprotective (M2) microglia in the ventral horn spinal cord of aged mice. This was placed in evidence by the increased proportion of microglial profiles displaying CD206 and P2Y12R, two molecules associated with the M2 phenotype, [82, 88, 89], in spinal cords of GTE-supplemented mice. The elevated presence of P2Y12R-positive cells with GTE supplementation is particularly interesting, since the purinergic receptor P2Y12 has been shown to be exclusively expressed in microglia and not in other infiltrating myeloid cells, and appears to be critical for microglial chemotaxis and extension of processes in response to injury [141]. In normal conditions, P2Y12R-positive microglia is a relative stable cell population throughout the lifespan, and this stability seems to be important to ensure the homeostatic functions of central nervous system during aging [142]. P2Y12R expression has been reported to decline in hippocampal demyelinating lesions of aged mice [143] and in multiple sclerosis patients [142], but increases in human brain after anti-inflammatory stimuli [88, 89]. In line with these results and in agreement with the anti-inflammatory and anti-oxidative actions of GTE, we found that P2Y12R-positive, neuroprotective M2, microglia significantly increases after chronic intake of GTE and translocate P2Y12R to the nucleus. The nuclear expression of this purinergic receptor in microglia following GTE supplementation is difficult to conciliate with the role of a G-protein coupled receptor such as P2Y12R, considered as a cell-surface receptor whose primary function is to transduce extracellular stimuli into intracellular signals [144]. Nevertheless, our results are in concordance with previous data on microglial activation in gliomas, in which it has been reported a shift from cytoplasmic to nuclear expression of P2Y12R linked to the acquisition of the M2 phenotype [90]. The functional consequences of this nuclear translocation of P2Y12R are not unraveled yet. In any case, the increase of neuroprotective M2 microglia we found correlates with the prevention of MN deafferentation observed in aged animals subjected to dietary supplementation. However, more work is necessary to analyze whether GTE- intake results in a direct action on microglia, dampening their anti-inflammatory phenotype or, conversely, flavonoids have their primary effect stabilizing MN afferent inputs, this resulting in the preclusion of microglial reaction.

The effects of supplementation with cocoa flavanols on age-associated microglia activation found here are striking. Although cocoa did not reduce microgliosis in ventral horn of aged animals, it significantly decreased the density of activated/phagocytic, CD68-positve, microglia located close to MNs. Microglia activation appears to be necessary for the removal of VGluT1 synapses on MNs, as reported following peripheral nerve injury [145]. In this regard, we have previously shown that microglia expressing CD68 are involved in the phagocytosis of VGluT1 nerve terminals in ventral horn of SMA mice [118]. Mechanisms underlying the elimination of afferent nerve terminals contacting MNs by microglia in pathological conditions are still not well clarified; it seems likely, however, that not all types of synaptic contacts on MNs are eliminated in the same way by microglia. Thus, it is tempting to speculate that, during the neuroinflammatory response occurring in the ventral horn with aging, the removal of VGluT1 nerve terminals contacting MNs requires specific mechanisms different from those operating to eliminate VAChT or VGluT2 synapses. In fact, in a previous work, we have reported that after peripheral nerve transection, activated microglial cells are recruited to injured MNs, with a predominant positive chemoattraction to VGluT1 and less to VAChT synapses, but with minor preferences for contacting other synapse types, such as GABAergic and serotoninergic boutons [146]. Accordingly, it is plausible that activated microglia remove VGluT1 synapses more easily and in a higher extent than VAChT and VGluT2 boutons. This would explain the differential effect on preventing MN deafferentation observed with GTE- and cocoa-supplementation and why dampening microglial activation (indicated by the reduced density of CD68-positive profiles) in areas surrounding MNs following cocoa supplementation precludes VGluT1, but not VAChT and VGluT2, synapse loss.

## CONCLUSIONS

**Figure 10:**
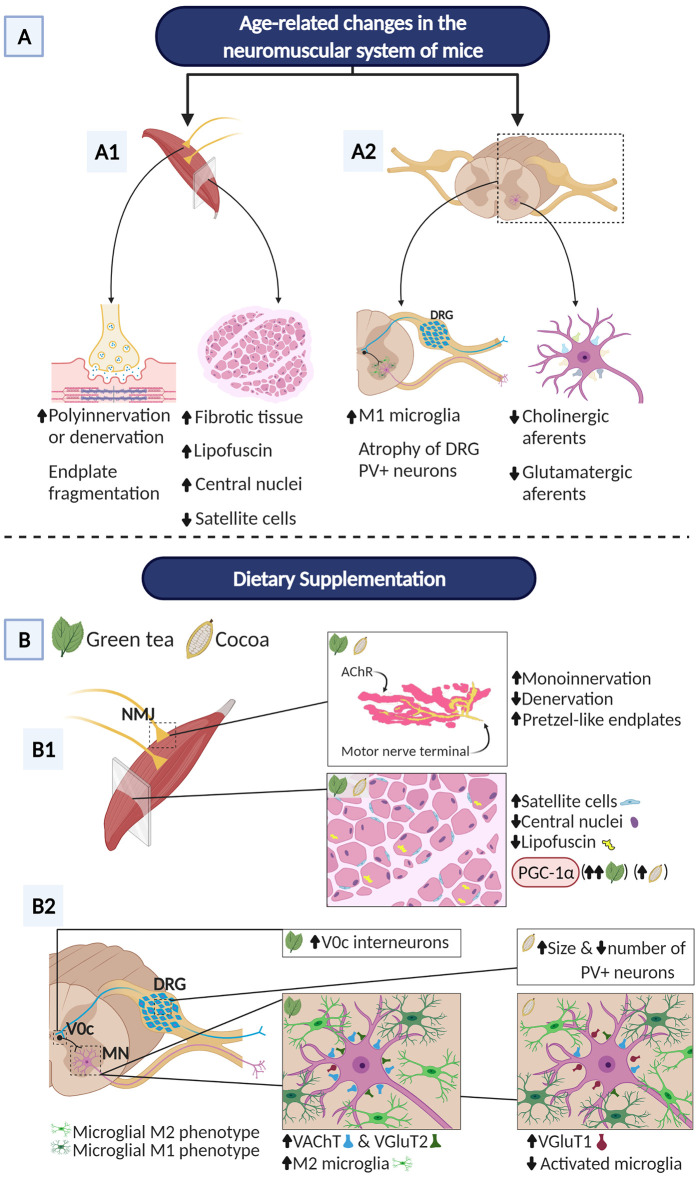
**Overview of main benefits promoted by GTE and cocoa dietary supplementations on aging-associated changes in the neuromuscular system of C57BL/6JRj mice.** The hallmark neuromuscular alterations occurring in these mice in the course of aging [6] are also summarized. (**A**) NMJs of aged mice display signs of either denervation or polyinnervation, and endplate fragmentation, suggesting an active process of NMJ remodeling and muscle reinnervation. Additionally, aged muscles show increased fibrosis, abundant fibers with lipofuscin accumulation and centrally located nuclei (indicative of muscle regeneration), and a marked reduction in the proportion of SCs. Aged mouse spinal cords exhibit reactive gliosis in ventral horn with increased proportion of harmful M1 microglia and significant loss of excitatory cholinergic (C-boutons) and glutamatergic synapses on MNs; atrophy of sensory proprioceptive (PV-positive) DRG neurons was also seen. (**B**) GTE and cocoa supplementations significantly decrease muscle denervation and signs of NMJ degeneration; both supplements augment the proportion of NMJs exhibiting single innervation, reduce fragmentation of endplates and increase the number of them exhibiting a healthier, “pretzel-like”, appearance. Furthermore, GTE- and cocoa-enriched diets increase the density of satellite cells, and reduce lipofuscin deposition in myofibers and the proportion of them displaying central nuclei. PGC-1α, a key regulatory factor of mitochondrial biogenesis, shows increased muscular levels in animals fed with GTE and cocoa-supplemented diets. GTE-, but not cocoa-, supplementation prevents the aging-associated loss of cholinergic (C-bouton) and VGluT2-positive glutamatergic synapses on lumbar spinal cord MNs; cocoa, but not GTE, increases the density of VGluT1-positive glutamatergic nerve terminals contacting MNs. The prevention of age-related C-bouton loss promoted by GTE is associated with increased numbers of V0_C_ interneurons, the neuronal origin of cholinergic C-bouton inputs to MNs. Additionally, the prevention of aging-associated loss of VGluT1-positive MN-afferents by cocoa is accompanied by the increased body size of PV-positive proprioceptive DRG neurons, the source of Ia VGluT1 afferents to MNs. Moreover, GTE-supplementation improves age-related reactive microgliosis in the spinal cord and increases the proportion of neuroprotective M2 microglial cells around MNs, indicating that the imbalance of M1/M2 microglia found to occur with aging can be potentially modulated by GTE. Created with BioRender.com.

In summary, dietary intake of flavonoids from green tea or cocoa was able to significantly increase the survival rate of aged mice and to prevent some regressive structural changes occurring with senescence in distinct cellular components of the neuromuscular system (Figure 10). Both diets clearly preserved NMJ innervation and maturity, delayed the senescence process of the skeletal muscle, and enhanced its regenerative capacity, as inferred from the more “youthful” cellular phenotype of myofibers, the apparent reduction of myofiber degeneration/regeneration cycles, the preservation of the myogenic SC population, and the increased expression of PGC-1α. Additionally, GTE- and cocoa-flavonoids differentially promoted the stability of excitatory synaptic inputs to MN, alleviate microgliosis and modulate the balance between proinflammatory (M1) and neuroprotective (M2) microglial phenotypes. The differential effects of flavonoids from GTE and cocoa on changes occurring in the spinal cord with age, suggest that there might be a benefit to combine different flavonoids to counteract the negative effects of aging in the neuromuscular system. Furthermore, the association of flavonoid supplementations with regular physical exercise must be taken in consideration in the therapy of sarcopenia. Future research is needed to investigate whether higher doses of flavonoids are needed and/or longer-term interventions can help restore proper motor function. In any case, the results shown here provide insight into the use of flavonoids as nutritional interventions for the treatment of aging-related neuromuscular alterations that occur with sarcopenia. Our data should be also considered in the context of the dietary management of patients affected by muscular and MN diseases.

## MATERIALS AND METHODS

### Animals and dietary regimens

Male C57BL/6JRj mice were obtained from Janvier Labs (Saint Berthevin, France), housed in a standard cage (3 animals per cage) containing a nest box (Smart-House, ZOONLAB GmbH, Castrop-Rauxel, Germany), and maintained in a 12-h light/dark cycle, and controlled room temperature (20 ± 2° C) and humidity (50%). A total of 45 mice (22 month-old) were randomly distributed in 3 groups (n = 15 per group) and fed with either: a standard AIN-93M diet (control group), the AIN-93M diet supplemented with 0.5% GTE (Suphenon 90D, Taiyo, GTE group), or the AIN-93M diet supplemented with 0.133% high-flavanol cocoa powder (Acticoa, Barry Callebaut, cocoa group). The GTE group was designed to receive an average dose of ~200 mg EGCg/Kg body weight/d. The cocoa group was designed to receive an average dose was ~13.33 mg flavanols/kg body weight/d. Diets started at week 92 (21 months) and were maintained until week 116 (27 months). Daily food intake was measured across groups. Animals were allowed ad libitum access to drinking water and to either the control, or GTE- or cocoa-supplemented diets. Feed intake was estimated by subtracting the weight of the remaining food from that of the food administered the week before. For some analyses, adult (4 month-old) C57BL/6JRj mice, feed with the standard diet, were also used.

All animal handling and experimentation procedures were conducted in accordance with the ethical standards laid down in the 1964 Declaration of Helsinki and its later amendments, and the European Communities Council Directive 2010/63/EU for the care and use of laboratory animals and the norms established by the Generalitat de Catalunya (published in the Diari Oficial de la Generalitat de Catalunya [DOGC] 2073, 1995). All experiments were previously evaluated and approved by the Committee for Animal Care and Use of the Universitat de Lleida, and the Committee for Ethics on Experimental Animal and Human Research of the Universitat Autònoma de Barcelona. All efforts were made to minimize suffering and reduce the number of animals in agreement with the European Communities Council Directive (November 24, 1986; 86/609/EEC). According to previous defined criteria [147, 148], mice displaying tumors, physical abnormalities and/or evidence of disease were excluded from the study and euthanized by an overdose of pentobarbital (30 mg, intraperitoneally).

### Motor behavior tests

To evaluate the impact of different diets on motor skills, mice were periodically weighed and carefully examined. Open-field (for assessing locomotor activity), and pen (balance beam, for assessing motor coordination and balance) tests were subsequently performed every 4 weeks according to previously described guidelines [149]. Tests were conducted by the same investigator in a blinded manner. In the pen test, every mouse was evaluated 3 times, with a 15-minutes recovery period between them; the value obtained in the best test performance was used as the final score. Open-field test was performed by the automated recording of mouse movements using Smart Video Tracking software (v2.5.21, Panlab Harvard Apparatus, Holliston, MA, USA); different parameters such as time, distance, entries in zones, and average speed were measured.

### Electrophysiology tests

For motor nerve conduction tests, the sciatic nerve was stimulated percutaneously by means of single pulses of 25 μs duration delivered through a pair of needle electrodes placed at the sciatic notch. The CMAP (M wave) and the H-reflex wave were recorded from TA and plantar interossei (PL) muscles by using microneedle electrodes [150]. The H/M ratio was calculated as the quotient of the maximal H wave and maximal M wave amplitude for each recorded muscle. For sensory nerve conduction tests, the recording electrodes were placed near the digital nerves of the fourth toe, recording the compound sensory nerve action potential (SNAP) following stimulation of the sciatic nerve as above. Signals were amplified and displayed on a digital oscilloscope (Tektronix 450S, Tektronix, Beaverton, OR, USA) at appropriate settings to measure the amplitude from baseline to the maximal negative peak, and the latency from stimulus to the maximal negative peak. To ensure reproducibility, the recording needles were placed under microscope guided by anatomical landmarks to secure the same placement on all animals. During the tests, the mice body temperature was maintained constant between 34-36° C by means of a thermostat-controlled heating pad.

MUNE test was performed in the TA muscle, using the same setting as for the motor nerve conduction test. The protocol used was based on the incremental technique [151]. Starting from subthreshold intensity, the sciatic nerve was stimulated with single pulses of gradual increased intensity until the first CMAP response appeared, representing the first motor unit recruited. With the next stimuli, quantal increases in the response were recorded. Increments >50 μV were considered as the recruitment of an additional motor unit. The amplitude of a single motor unit action potential (SMUA) was calculated as the mean of more than 15 consistent increases. The estimated number of motor units resulted from the equation: MUNE = CMAP maximal amplitude/mean amplitude of SMUA.

### Tissue sample collection and histological analysis

Mice were anaesthetized and transcardially perfused with 4% paraformaldehyde (PFA) in 0.1 M phosphate buffer (PB), pH 7.4. After animal perfusion, spinal cords, L4 DRGs, L4 VRs, and entire individual hindlimb skeletal muscles were rapidly dissected from each mouse. VRs were then immersed in 1% PFA and 1% glutaraldehyde in 0.1 M PB (pH 7.4) for 24 hours.

Muscle samples were processed for histology and further morphometry according to previously described procedures [152]. Muscles examined were the TA, a fast-twitch muscle, which helps with dorsiflexion and inversion of the foot, and the Sol, a relative slow-twitch muscle in mice, which plays an important function in maintaining the standing posture and is also used for plantarflexion during walking [153]; to examine whether some changes also occurred in a more proximal hindlimb muscle, the fast-twitch Gra muscle, which is mainly involved in the adduction of the limb and knee flexion [153], was also included in some experiments. Muscles were cleaned, cleared of the excess of connective tissue, blotted dry and weighed. After this, they were post-fixed in 4% PFA in 0.1 M PB (pH 7.4), cryoprotected with 30% sucrose in 0.1 M PB, embedded in Tissue Freezing Medium (Triangle Biomedical Sciences, Durham, NC) and frozen. Some cryostat transverse sections (16-μm thick), obtained from the mid-belly of the muscle, were stained with hematoxylin and eosin for histological examination.

VRs were post-fixed in 1% osmium tetroxide, and embedded in Embed 812 (Electron Microscopy Sciences, Fort Washington, PA) epoxy resin, following standard procedures. Semithin transversal sections (1-μm thick) were stained with methylene blue and imaged using an Olympus 60x/1.4NA PlanApo oil immersion objective (Olympus) and a DMX 1200 Nikon (Tokyo, Japan) digital camera.

### Immunohistochemistry and imaging

For immunohistochemical analysis, lumbar spinal cords, L4 DRGs and skeletal muscles were post-fixed by immersion in 4% PFA in 0.1M PB (pH 7.4), either overnight (for spinal cords and DRGs) or for 2 hours (for muscles), and cryoprotected. Tissue samples were embedded in tissue freezing medium and frozen. Longitudinal (16-μm thick, for muscles) and transverse (16-μm thick, for muscles, and 14-μm thick, for spinal cords and DRGs) serial cryostat sections were obtained and stored at -80° C.

Tissue sections were sequentially rinsed in phosphate-buffered saline (PBS) containing 0.1% Triton X-100 for 30 min, blocked in normal goat serum or normal horse serum, and subsequently incubated with the chosen primary antibody. The primary antibodies used are indicated in Table 1.

**Table 1:** Primary antibodies used for immunohistochemistry.

**Target**	**Abbreviation**	**Host species**	**Source /catalog no.**	**Dilution**
Agrin	Agrin	mouse monoclonal	Millipore (Temecula, CA) / MAB5204	1:100
ATP5A	ATP5A	mouse monoclonal	Abcam (Cambridge, UK) / ab14748	1:100
Calcitonin gene-related peptide	CGRP	rabbit polyclonal	Sigma-Aldrich (Madrid, Spain) / C8198	1:1000
Macrophage Mannose Receptor	MMR/CD206	goat polyclonal	R&D Systems (Minneapolis, MN) / AF2535	1:100
Cluster differentiation 68	CD68	rat monoclonal	Bio-Rad Laboratories Inc. (Hercules, CA) / MCA1957	1:100
Growth associated protein 43 (H-100)	GAP-43	rabbit polyclonal	Santa Cruz Biotechnology (Dallas, TX) / sc-10786	1:50
Ionized calcium-binding adaptor molecule 1	Iba 1	rabbit polyclonal	Wako Pure Chemical Industries Ltd. (Osaka, Japan) / 019-19741	1:500
Ionized calcium-binding adaptor molecule 1	Iba 1	goat polyclonal	Abcam / ab5076	1:500
Laminin 2	Laminin 2	rat monoclonal	Sigma-Aldrich / L0663	1:100
Mac-2/Galectin 3	Mac-2	rat monoclonal	Cedarlane (Burlington, Canada) / CL8942AP	1:800
Neurofilament 68 KDa	NF	chicken polyclonal	Abcam / ab72997	1:1000
G-protein-coupled purinergic receptor P2Y12R	P2Y12R	rat monoclonal	Biolegend (San Diego, CA) / 848001	1:100
Paired box protein 7	Pax7	mouse monoclonal	R&D Systems / MAB1675	1:50
Parvalbumin	PV	mouse monoclonal	Swant (Marly, Switzerland) / PV235	1:2000
Synaptic vesicle protein 2	SV2	mouse monoclonal	DSHB / SV2	1:1000
Vesicular acetylcholine transporter	VAChT	guinea pig polyclonal	Synaptic Systems (Gottingen, Germany / #139105	1:500
Vesicular glutamate transporter 1	VGluT1	rabbit polyclonal	Synaptic Systems / #135302	1:1000
Vesicular glutamate transporter 2	VGluT2	guinea pig polyclonal	Synaptic Systems / #135404	1:200

After incubation with the primary antibody, sections were washed with PBS and incubated for 1 hour at room temperature (RT) with the appropriate secondary antibodies labeled with one of the following fluorochromes (1:500): Alexa Fluor 488, Alexa Fluor 546, (Molecular Probes, Eugene, OR, USA), Cy3, or Cy5 (Jackson Immuno Research Laboratories, West Grove, PA, USA). The spinal cord sections were finally counterstained with blue fluorescent NeuroTrace Nissl staining (1:100; Molecular Probes). Muscle sections were incubated with Alexa Fluor 555-conjugated α-Bgtx (1:500; Molecular Probes) to identify postsynaptic acetylcholine receptors. Some sections were also stained with 4’,6-diamidino-2-phenylindole dihydrochloride (DAPI, 50 ng/ml, Molecular Probes) for DNA staining. To avoid lipofuscin-like autofluorescence, slides with sections of spinal cord from old mice were treated with the Autofluorescent Eliminator Reagent (Merck, Kenilworth, NJ, USA), following incubation with the secondary antibodies. Some slides with DRG were stained with FITC-conjugated IB4 (1:25; Sigma-Aldrich).

After washing, slides were coverslipped using an anti-fading medium containing 0.1 M Tris-HCl buffer (pH 8.5), 20% glycerol, 10% Mowiol, and 0.1% 1,4-diazabicyclo[2,2,2]octane. Slides were examined with an Olympus BX51 epifluorescence microscope (Olympus, Hamburg, Germany) equipped with a DP30BW camera, or with FluoView 500 or FluoView 1000 Olympus laser scanning confocal microscopes. For comparisons, slides from different animals and experimental conditions were processed in parallel for immunohistochemistry and subsequent imaging. The same scanning parameters were used for the acquisition of images corresponding to different experimental groups. For spinal cord immunohistochemical studies, digital images of the entire lumbar region were obtained from every 30^th^ section.

### Image analysis and morphometry

Image and morphometric examinations were performed by two independent investigators. Digital images were analyzed with ImageJ software (US National Institutes of Health, Bethesda, MD, USA).

Myofiber cell counts and size measurements were performed in a single random image per muscle taken from its mid-belly and immunostained for laminin. The proportion of myofibers showing central nuclei was also counted on images from DAPI-stained sections. The percentage of myofibers showing lipofuscin aggregates, the number and the area of these aggregates, and the area and intensity of ATP5A immunostaining, used as mitochondrial marker, were also measured by ImageJ. The number of DAPI-stained nuclei exhibiting Pax7-positive immunolabeling, as a marker of satellite cells, was also counted in confocal images of muscle sections.

The cytoarchitecture of the NMJs was assessed in longitudinal immunostained sections of muscles. Z-stack optical sections (1-μm thick) were obtained with the confocal microscope and projected to reconstruct NMJs; maximum intensity projections of stacks were created using the microscope software. Three-five sections were examined for each muscle, in which NMJs from different randomly selected visual fields were analyzed. NMJ size was measured by determining the area of manually outlined α-Bgtx-labeled postsynaptic site. Full or partial denervation was considered when the postsynaptic site was not completely apposed by the nerve terminal immunostained for both NF and SV2, as presynaptic marker: a NMJ was considered to be denervated when the percentage of α-Bgtx-labeled postsynaptic surface covered by SV2-stained presynaptic terminal was <15%. Single or poly-innervation was estimated by counting the number of axon terminals, stained with anti-NF+SV2 antibodies, entering a single postsynaptic site: an NMJ was defined as polyinnervated when it was occupied by two or more axons. Terminal sprouting was quantified by counting the number of NF+SV2-stained axonal processes coming from a nerve terminal that escaped from a α-Bgtx-labeled postsynaptic site; the number of NMJs showing sprouts was referred as a percentage of the total number of NMJs examined per muscle. On the basis of the morphological appearance of the postsynaptic site, the degree of NMJ maturity was classified as plaque, folds, perforations, or pretzel-like (secondary) structure ranging from immature to mature, according to previously described criteria [154]. An NMJ was defined as fragmented when its postsynaptic site displayed a discontinued appearance, with 5 or more AChR-islands stained with α-Bgtx, and/or unusually small and irregular clusters of AChR. Immunostaining for CGRP, GAP-43 and agrin was estimated based on pixel intensity after background subtraction in NMJs which postsynaptic site was visualized with α-Bgtx. An average of 50 NMJs per muscle was examined to assess the different morphometric parameters and the intensity of immunostaining. Any NMJ that was difficult to examine due to its location and/or orientation was excluded from the analysis. An average of 35 muscle sections from 3-5 animals per condition were analyzed.

The analysis of afferent synapses on MNs and glia in the ventral horn was performed on confocal images taken from every 30^th^ section of the entire lumbar spinal cord. The number and area of VAChT-, VGluT1- and VGluT2-immunoreactive synaptic boutons on MN somata were counted and measured by delineating their periphery on the screen; only boutons that were in close contact with MNs displaying a large nucleus and visible nucleolus were included in the counts, and numbers were normalized to the perimeter of MN soma. Levels of immunoreactivity for Iba1, Mac2, CD206, P2Y12R and CD68 in the spinal cord were quantified by analyzing pixel intensity following background subtraction. Spinal cord microglia morphology was quantified in confocal images from Iba1 immunostained sections by using ImageJ software for skeleton analysis (AnalyzeSkeleton [2D/3D] from http://imageJ.net/AnalyzeSkeleton). Analysis was performed according to a previously described procedure [80]. For this, digital photomicrographs were transformed to 8-bit grayscale, and then binarized to obtain a black and white image by means of a formerly established threshold. Every image was manually edited to obtain a continuous set of pixels and gaps between processes belonging to neighboring cells. The image was then saved and the plugin AnalyzeSkeleton(2D/3D) run. The accuracy of skeletonized images was assessed, by creating an overlay of the obtained skeleton with the corresponding original image.

The density of V0_C_ neurons in lumbar spinal cord was measured by counting on digital images the number of VAChT-immunolabeled interneurons in a 0.1 mm^2^ area around the central canal where these cells are placed.

The number and soma area of CGRP, IB4 and PV-immunolabeled sensory neurons in DRGs were also measured on digital images. The number and diameter of healthy and degenerating myelinated axons were determined on 60x images taken from VR semithin cross-sections stained with methylene blue. The images were joined together to obtain a whole picture of an entire nerve root transverse section. The diameter of the myelinated axons was measured by delineating the outer profile of the myelin sheath. Axon diameter and *g*-ratio measurements were performed on micrographs of VRs; ImageJ *g*-ratio plug-in was used to obtain semiautomated measurements of randomly selected nerve fibers; the axon diameter was divided by the outer diameter of the myelin sheath. At least 100 myelinated axons per mouse (4-5 mice per condition) were measured.

### Western blotting

Frozen hindlimb muscles were fragmented and homogenized using an electric homogenizer with ice-cold RIPA lysis buffer (50 mM Tris-HCl [pH 7.4], 150 mM NaCl, 1 mM EDTA, 1% NP-40, 1% Na-deoxycholate, 0.1% SDS) supplemented with protease inhibitor (Sigma-Aldrich, cat. # P8340) and PhosSTOP (Roche, Laval, Canada). The homogenized samples were centrifuged at 13,000 rpm for 20 min at 4° C. The protein concentrations of supernatants were determined by BIO-RAD Micro DC protein assay (BIO-RAD, Laboratories Inc., Hercules, CA, USA). Loading buffer 4 x SS (20% sucrose and 0.05% bromophenol blue, 0.1% sodium azide) containing 5-10% β-mercaptoethanol (Sigma-Aldrich) and 15-50 μg of protein were loaded in either 7.5% or 10% polyacrylamide electrophoresis gel. Proteins were electrotransferred to polyvinyldifluoride membranes (ImmobilonTM-P, Millipore) in Tris-glycine-methanol-buffered solution. Membranes were blocked with 5% dried skim milk in 0.1% Tween 20 and Tris-buffered saline pH 8 (TBST) for 1 hour at RT, and then extensively washed in TBST. Immunodetection was performed by incubating the membranes overnight at 4° C with rabbit polyclonal anti-PGC-1α (1:500; Santa Cruz Biotechnology, cat. # sc-13067), rabbit polyclonal anti-GAP-43 (diluted 1:1000; Santa Cruz Biotechnology cat # sc-10786), mouse monoclonal anti-α-tubulin (diluted 1:1000; Sigma-Aldrich; cat. # T5168), and mouse monoclonal anti-glyceraldehyde 3-phosphatase dehydrogenase (GAPDH, diluted 1:10,000; Abcam, cat. # ab8245) antibodies, the two latter used for loading controls. The membranes were washed in TBST, incubated with the appropriate peroxidase-conjugated secondary antibodies (1:10,000; Amersham Biosciences, Buckinghamshire, UK) for 60 minutes at RT, washed in TBST and visualized using the ECL Prime Western Blotting Detection Reagent detection kit (GE Healthcare, Buckinghamshire, UK), as described by the manufacturer. The quantification of band densities was performed by using Chemi-Doc MP Imaging System (BIO-RAD Laboratories Inc.).

### Statistical analysis

Data are shown as the mean ± standard error of the mean (SEM). The statistical analysis was performed by either one-way or two-way analysis of variance (ANOVA) followed by *post hoc* Bonferroni’s test or two-tailed Student’s *t*-test when only two different groups were compared. Gehan-Breslow-Wilcoxon test was used for comparing survival curves between groups. The level of significance was established at *p* ≤ 0.05. GraphPad Prism 6 software was used for statistical analysis and graph presentations of data.

## Supplementary Material

Supplementary Figures

Supplementary Table 1
