# Supplementary material for: Beneficial effects of dietary supplementation with green tea catechins and cocoa flavanols on aging-related regressive changes in the mouse neuromuscular system
Source: Aging (Albany NY). 2021 Jul 28;13(14):18051–93. doi: 10.18632/aging.203336 (PMC8351677; doi:10.18632/aging.203336)
Supplement: Supplementary Figures [file aging-13-203336-s001.pdf]

SUPPLEMENTARY FIGURES

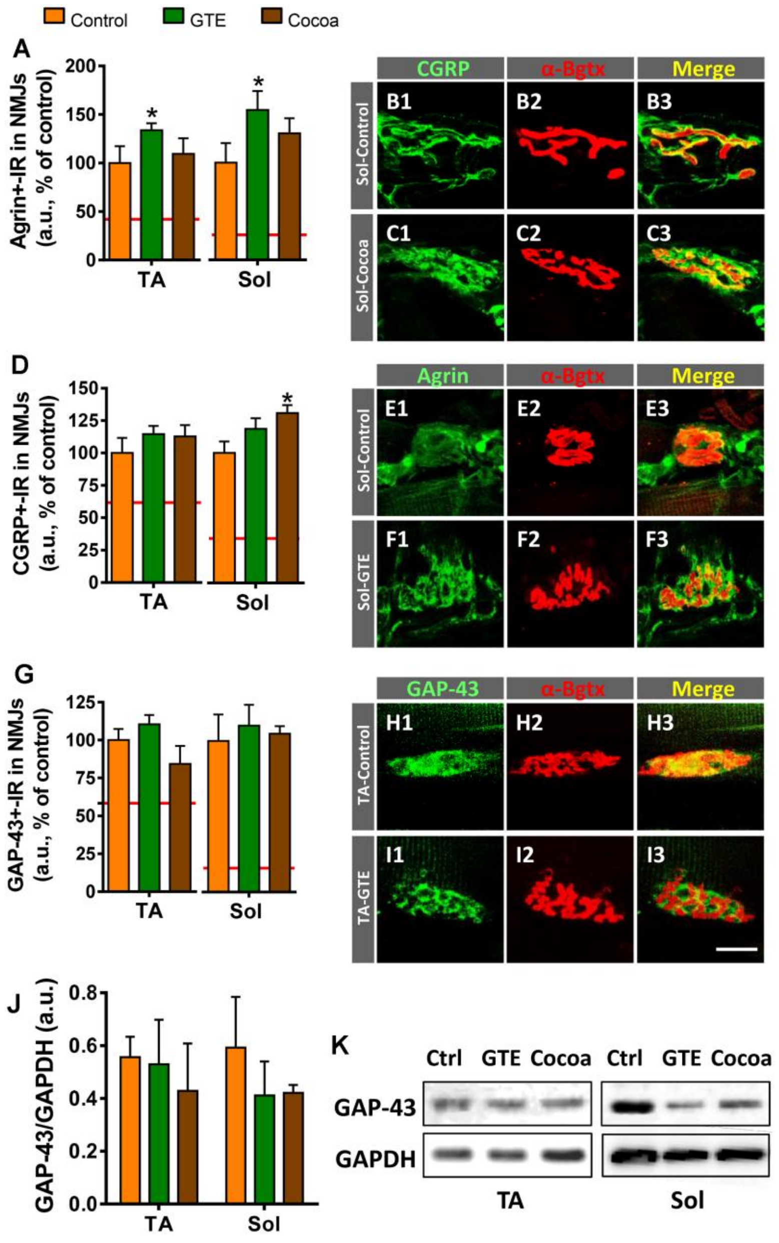

Supplementary Figure 1. Effects of GTE- and cocoa-supplemented diets on CGRP, agrin and GAP-43 expression in NMJs of aged muscles. The quantification of CGRP- (**A**) agrin- (**D**) and GAP-43- (**G**) immunoreactivity (IR, arbitrary units, [a.u.]) in NMJs of TA and Sol muscles from control, GTE and cocoa groups is shown. Data in the graphs are expressed as the mean ± SEM, and shown as the percentage of change compared with controls; two-way ANOVA, Bonferroni's; *post hoc* test 40-70 NMJs per muscle type were examined; one TA and Sol muscle per animal (3, control; 4 GTE; and 5 cocoa) were used for analysis. (**B1**–**C3**, **E1**–**F3**, **H1**–**I3**) Representative confocal micrographs of NMJs from Sol and TA muscle sections immunolabeled for either, CGRP, agrin or GAP-43 (all in green), as indicated; sections were also stained with α-Bgtx (red) for endplate visualization. Note the slightly, although non-significant increase in agrin-immunoreactivity at NMJs, particularly of Sol muscles, of animals fed with the GTE-supplemented diet. (**J**) Densitometric analysis of GAP-43 content in TA and Sol muscles of animals belonging to the three experimental groups; data were normalized to GAPDH. Bars represent the values (mean ± SEM) of 3 mice per condition from 2 independent western blot analysis; two-way ANOVA (Bonferroni's *post hoc* test). (**K**) Representative western blots of GAP-43 and GAPDH (as loading control) proteins in TA and Sol muscles from mice of control (Ctrl), GTE and cocoa groups. Scale bar in **I3** = 20 µm (valid for **B1**–**C3**, **E1**–**F3**, **H1**–**I2**).

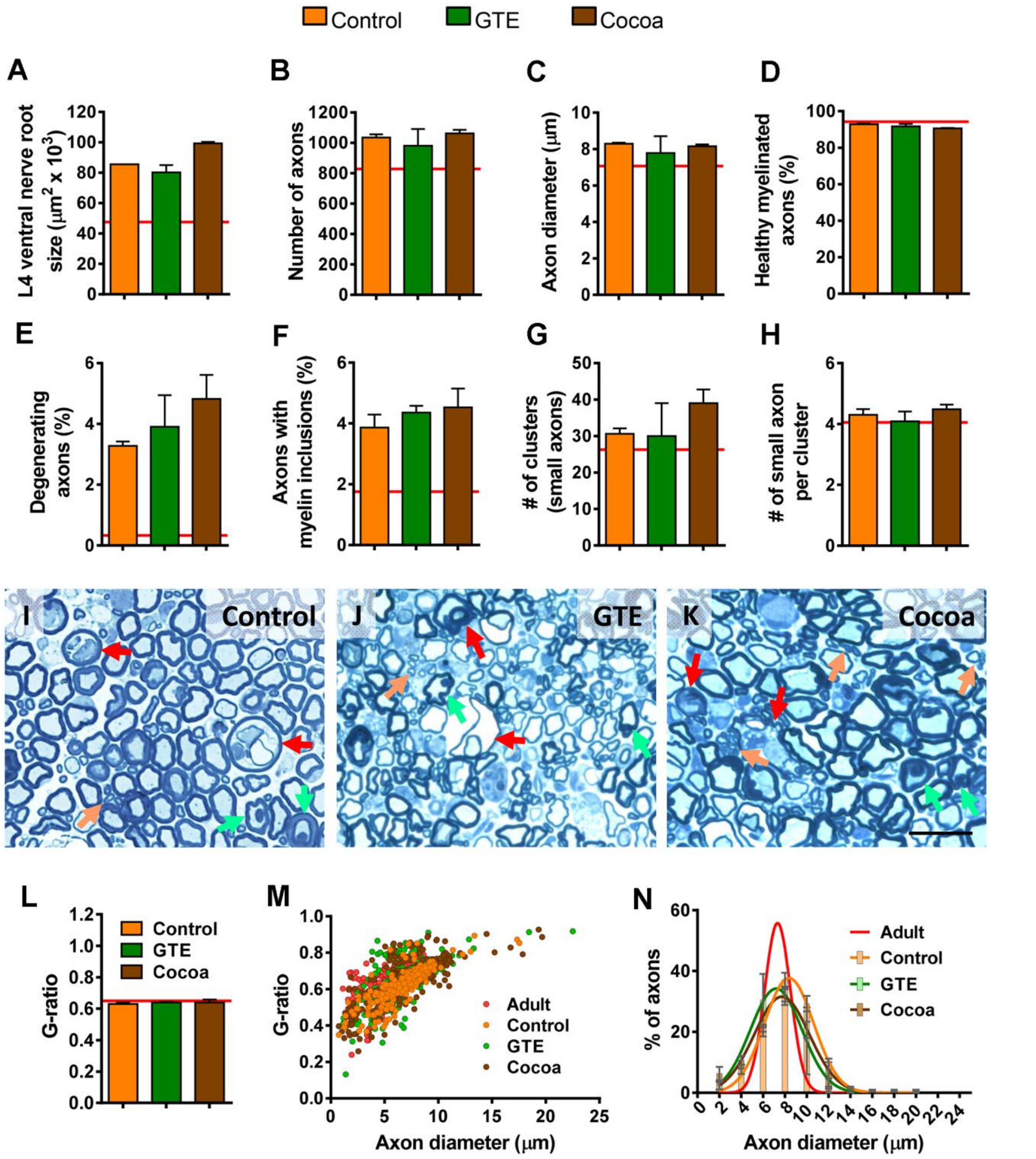

Supplementary Figure 2. Effects of GTE- and cocoa-supplementations in motor axons of aged mice. Analysis was performed in L4 VRs. (**A**–**C**) VR size (in µm^2, **A**), total number of axons (**B**) and axon diameter (including myelin sheath, in µm, [**C**]) in VRs. (**D**–**F**) Proportion of healthy myelinated axons (**D**), axons exhibiting a degenerate appearance (**E**), and those with myelin inclusions (**F**). (**G**, **H**) Number of small (≤6 µm diameter) axon clusters (≥3 axons) (**G**) and number of small axons per cluster (**H**) in VRs. (**I**–**K**) Representative images of methylene blue-stained semithin cross-sections of VRs from mice belonging to control (**I**), GTE (**J**) and cocoa (**K**) groups; arrows indicate: degenerating axons (red), axons with myelin inclusions (green) and clusters of small axons (orange). (**L**, **M**) Quantification of myelin thickness by *g*-ratio analysis (**L**) and scatter plot depicting *g*-rations in relation to axon diameter in (**M**) VRs. (**N**) Relative frequency of myelinated axon diameter (in µm); note the slight displacement to left of GTE and cocoa curves, indicative of a diameter reduction of motor axons of animals subjected to dietary flavonoid intake. Data in the graphs are expressed as the mean ± SEM of 3 VRs from different mice per condition. Scale bar in (**K**) = 25 µm (valid for [**I**] and [**J**]).

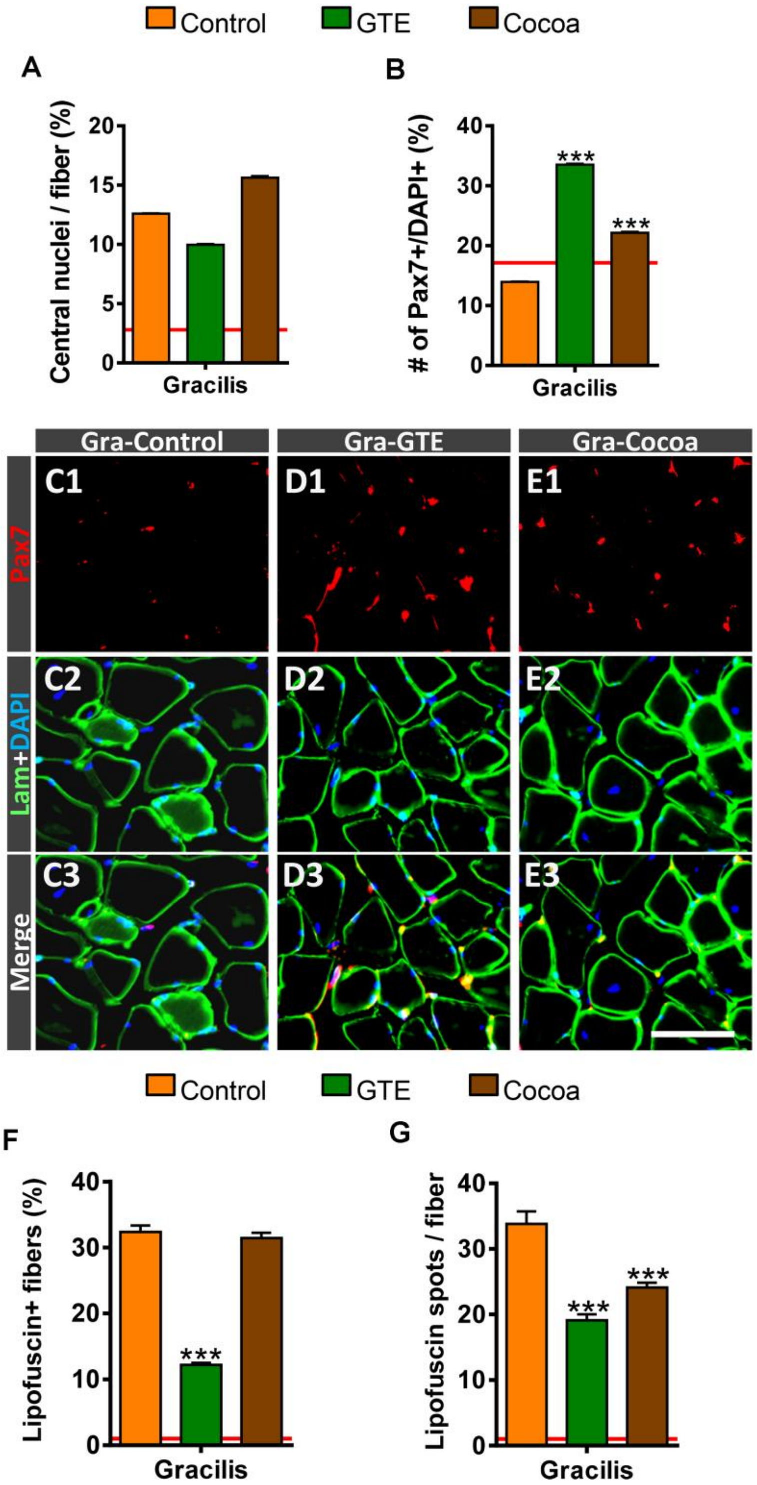

Supplementary Figure 3. Effects of GTE- and cocoa-supplemented diets on aging-associated changes in the proximal hindlimb Gra muscle. (**A**) Proportion of myofibers displaying central nuclei. (**B**) Percentage of Pax7-immunostained cells (SCs) with respect to DAPI-positive nuclei. Representative images of a combined immunolabeling for Pax7 (red) and laminin (green), and DAPI staining (blue) in transversal cryosections of Gra muscles from control, GTE and cocoa groups, as indicated. (**F**, **G**) Percentage of myofibers containing lipofuscin aggregates (**F**), and average number of lipofuscin granules per myofiber (**G**). Data in graphs are expressed as the mean ± SEM. The average values of different parameters analyzed in adult Gra muscles in a previous study [6] were indicated by a red line in each graph for comparative purposes. ****p* < 0.001 vs. control (Ctrl), two-way ANOVA, Bonferroni's *post hoc* test; sample size (myofibers): (**A**) = 1500-2500, (**B**) = 1700-2200, (**F**, **G**) = 1200-1700, from 3-5 mice per condition. Scale bars: 40 µm in **E3** (valid for **C1**–**E2**).

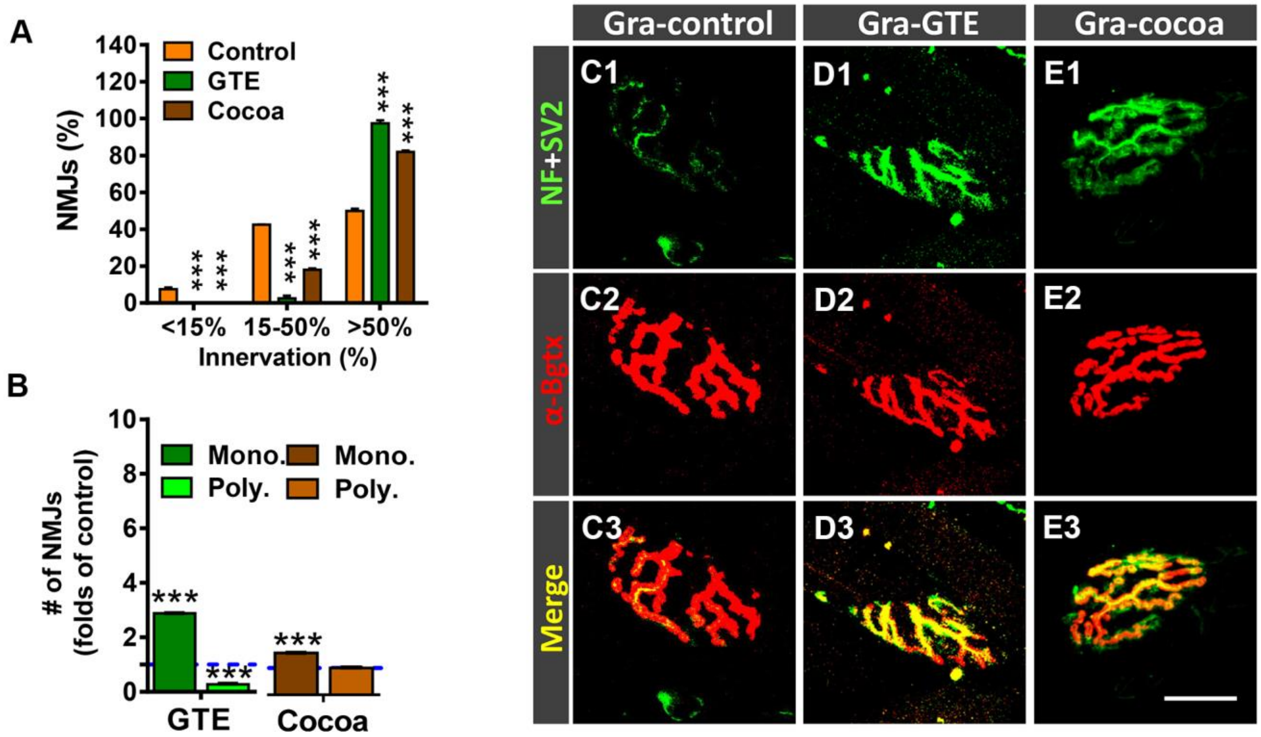

Supplementary Figure 4. Changes in the innervation degree of NMJs of Gra muscle in animals fed with GTE- or cocoa-supplemented diets. (**A**) Proportion of NMJs displaying different degrees of innervation; quantification was based on the percentage of α-Bgtx-labeled postsynaptic site area covered by SV2-immunostained presynaptic terminals (see Materials and Methods, <15% innervation was considered as denervated). (**B**) Number of NMJs exhibiting single (mono.) or multiple (poly.) innervation expressed as the percentage of control (blue dashed line). (**C1**–**E3**) Representative maximal projections of confocal stacks of NMJs from mice of control, GTE and cocoa groups (as indicated in panels); muscle sections were stained with antibodies against NF and SV2 (green, for presynaptic nerve terminals), and α-Bgtx (red, for postsynaptic AChR). Data in graphs are expressed as the mean ± SEM, ***$p < 0.001$ vs. control, one or two-way ANOVA, Bonferroni's *post hoc* test; sample size: 40-64 NMJs per muscle from 3-5 animals per condition. Scale bar in (**E3**) = 20 µm (valid for **C1**–**E2**).

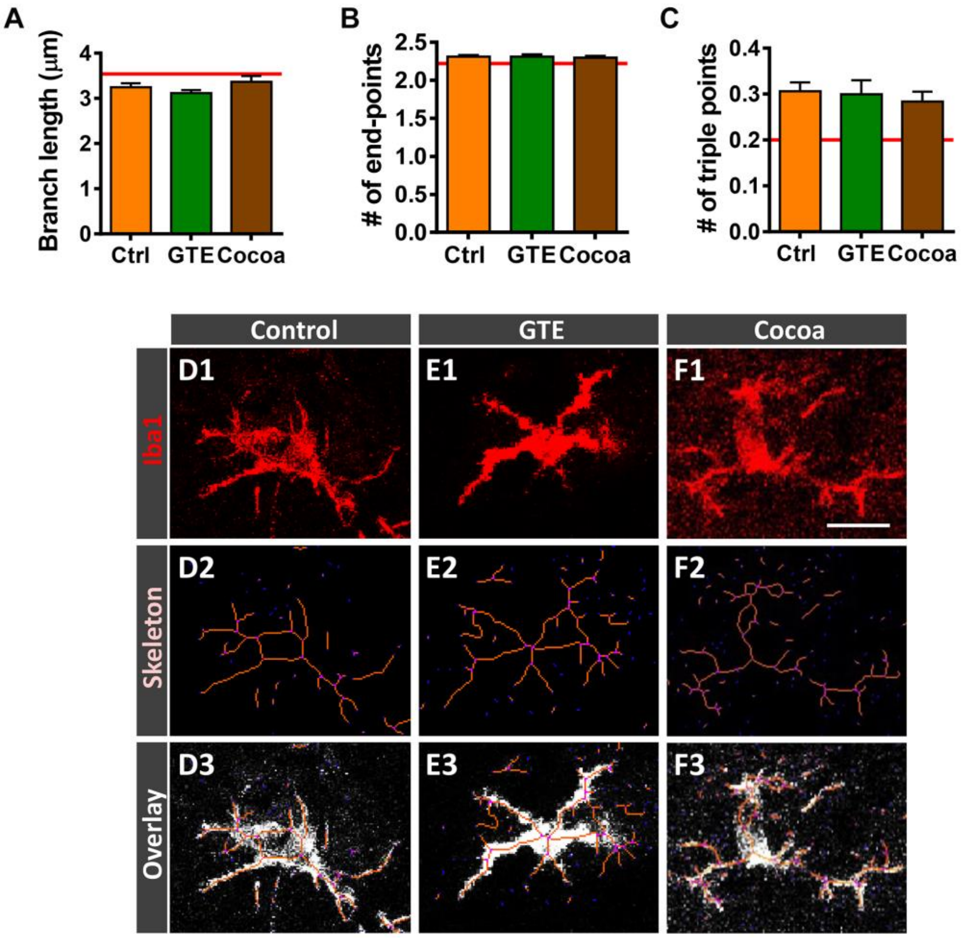

Supplementary Figure 5. Morphometric analysis of microglial complexity by using the skeleton approach (see Materials and Methods). The average branch length (in µm) (**A**), number of endpoints (**B**) and triple points (**C**) are shown in graphs. (**D1**–**F3**) Representative images showing Iba1-positive profiles (**D1**–**F1**), the skeletonized images (**D2**–**F2**), and the overlay of the skeleton and the original image of microglia (**D3**–**F3**) in control, GTE and cocoa groups as indicated in panels. Data in the graphs are expressed as the mean ± SEM; spinal cords of 3 control (Ctrl)-, 4 GTE- and 5 cocoa-group animals were used for analysis; a total of 50-130 images per experimental group were examined; the average values of the different parameters analyzed in adult animals [6] are shown in graphs as a red line for comparison. Scale bar in **F1** = 50 µm (valid for **D1**, **E1**).

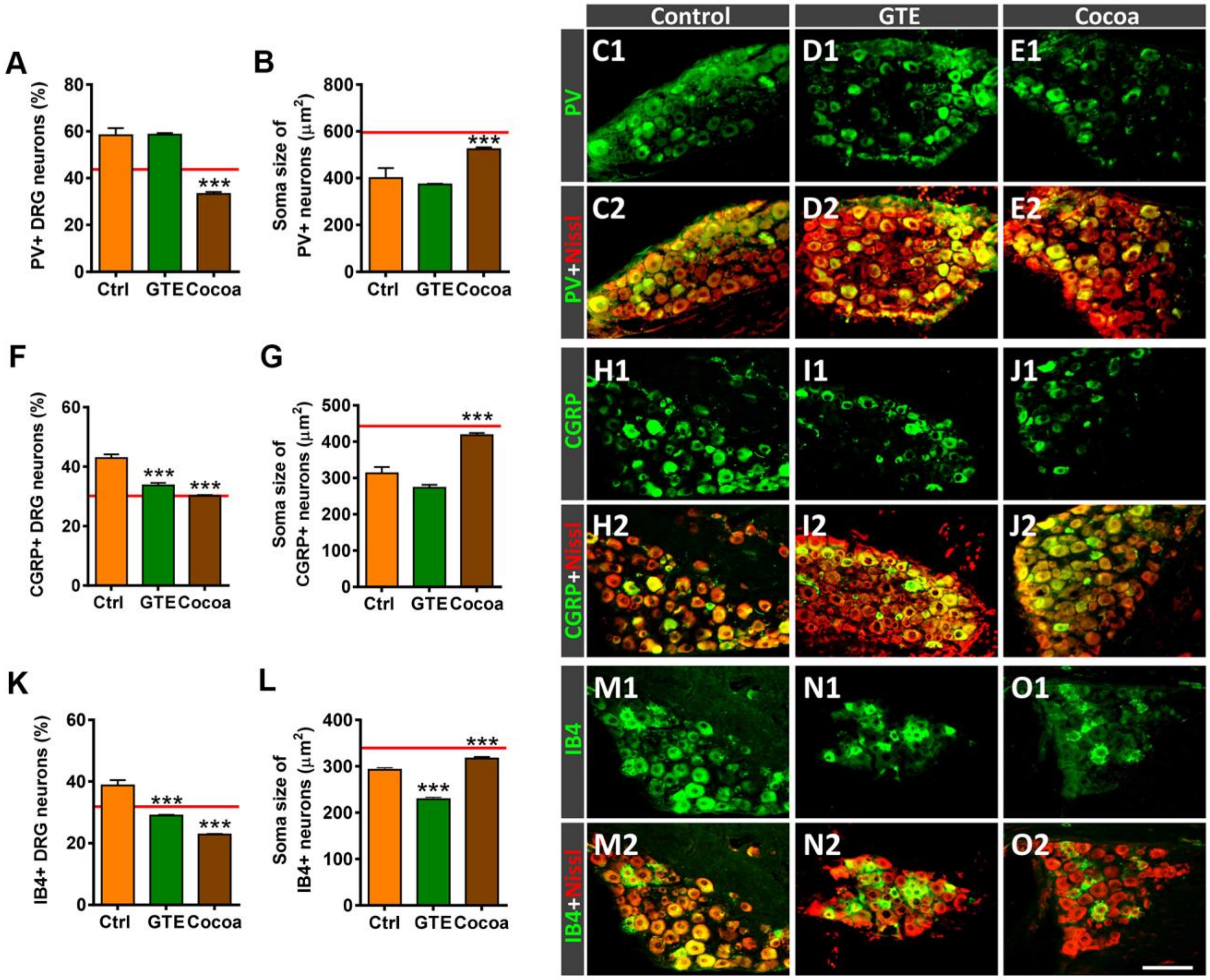

Supplementary Figure 6. Impact of GTE- and cocoa-supplemented diets in age-related changes in DRG sensory neurons. Proportion (**A**, **F**, **K**) and soma area (in μm^2, **B**, **G**, **L**) of neurons expressing PV (**A**, **B**) or CGRP (**F**, **G**), or stained with IB4 (**K**, **L**). (**C1**–**E2**, **H1**–**J2**, **M1**–**O2**) Representative micrographs obtained from DRG cryostat sections processed for PV, CGRP and IB4 staining (green) as indicated in panels; sections were also counterstained with fluorescent Nissl (red) for neuron visualization. Data in graphs are expressed as the mean ± SEM, ***p < 0.001 vs. control (Ctrl) (one-way ANOVA, Bonferroni's *post hoc* test); 30-80 images for each condition were analyzed; number of DRG examined: Ctrl =2, GTE = 3, cocoa = 5 from different animals; the red lines in graphs indicate the mean values in adult mice [6]. Scale bar: in **O2** = 100 μm (valid for **C1**–**E2**, **H1**–**J2**, **M1**–**O1**).

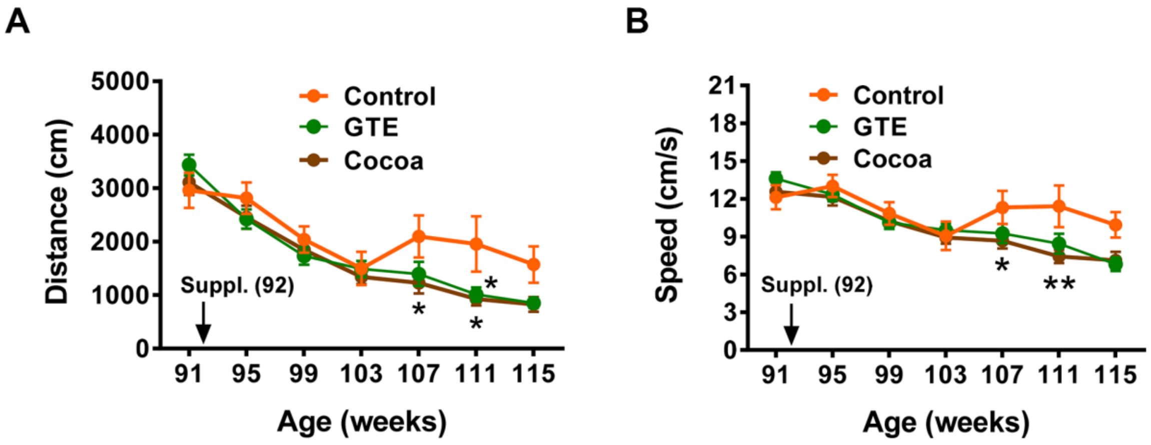

Supplementary Figure 7. Motor behavior analysis. Motor performance shown as the distance (in cm, **A**) and average speed (cm/s, **B**) assessed by the open-field test in mice from the three groups during the experimental period; the age-related decline in motor performance observed in the animals subjected to the standard (control) diet was not prevented by either GTE or cocoa supplementations; conversely, open-field parameters were even worsened in mice subjected to these dietary supplementations. Data are shown as the mean ± SEM (number of animals per group: 91 weeks of age, n = 15 in all groups; 107 weeks, control n = 8, GTE n = 13; cocoa n = 15; 114 weeks, control n = 6, GTE n = 12, cocoa n = 11); *$p < 0.05$ and **$p < 0.01$ vs. control; multiple *t*-test.
