# Supplementary material for: Beneficial effects of dietary supplementation with green tea catechins and cocoa flavanols on aging-related regressive changes in the mouse neuromuscular system
Source: Aging (Albany NY). 2021 Jul 28;13(14):18051–93. doi: 10.18632/aging.203336 (PMC8351677; doi:10.18632/aging.203336)
Supplement: Supplementary Table 1 [file aging-13-203336-s002.pdf]

## SUPPLEMENTARY TABLE

**Supplementary Table 1. Results from motor and sensory nerve conduction tests performed in old mice (24 months) fed with a standard (AIN-93M) diet (control), or the AIN-93M diet supplemented with either GTE- or cocoa flavanols.**

| Parameter | Old control (n=7) | Old GTE (n=5) | Old cocoa (n=5) |
|---|---|---|---|
| TA Lat peak (ms) | 1.55 ± 0.08 | 1.52 ± 0.03 | 1.48 ± 0.02 |
| TA CMAP (mV) | 50.5 ± 2.7 | 47.0 ± 4.9 | 53.4 ± 4.7 |
| SMUA (mV) | 1.20 ± 0.08 | 1.01 ± 0.04 | 1.18 ± 0.12 |
| MUNE | 40.8 ± 1.5 | 44.5 ± 2.6 | 44.9 ± 0.7 |
| PL Lat peak (ms) | 2.44 ± 0.19 | 2.64 ± 0.07 | 2.34 ± 0.10 |
| PL CMAP (mV) | 4.60 ± 0.55 | 4.22 ± 0.75 | 4.80 ± 0.58 |
| PL H wave Lat (ms) | 6.92 ± 0.40 | 7.51 ± 0.36 | 6.52 ± 0.21 |
| PL H/M ratio (%) | 32.1 ± 3.6 | 28.0 ± 8.8 | 17.8 ± 3.3 |
| DgN Lat (ms) | 1.39 ± 0.06 | 1.29 ± 0.07 | 1.32 ± 0.05 |
| DgN CNAP (µV) | 29.1 ± 1.8 | 31.0 ± 5.4 | 32.0 ± 4.7 |
| Weight (g) | 29.7 ± 0.7 | 25.1 ± 1.3 | 27.8 ± 0.9 |

TA, tibialis anterior muscle; PL, plantar interosseus muscle; DgN, 4th digital nerve; Lat, latency at the peak of the wave; CMAP, compound muscle action potential; CNAP, compound nerve action potential; MUNE, motor unit number estimation; SMUA, singer motor unit amplitude.

Statistical comparisons between groups were performed with one-way ANOVA (Bonferroni's *post hoc* test); no significant differences were found.
